# A unique approach toward near-infrared fluorescent probes for bioimaging with remarkably enhanced contrast†
†Electronic supplementary information (ESI) available: Synthetic procedures and characterization data; additional spectroscopic data. See DOI: 10.1039/c5sc04014k


**DOI:** 10.1039/c5sc04014k

**Published:** 2016-01-04

**Authors:** Yi-Jun Gong, Xiao-Bing Zhang, Guo-Jiang Mao, Li Su, Hong-Min Meng, Weihong Tan, Suling Feng, Guisheng Zhang

**Affiliations:** a Collaborative Innovation Center of Henan Province for Green Manufacturing of Fine Chemicals , Key Laboratory of Green Chemical Media and Reactions , Ministry of Education , School of Chemistry and Chemical Engineering , Henan Normal University , Xinxiang , Henan 453007 , P. R. China . Email: zgs6668@yahoo.com; b State Key Laboratory for Chemo/Biosensing and Chemometrics , College of Chemistry and Chemical Engineering , Hunan University , Changsha 410082 , China . Email: xbzhang@hnu.edu.cn

## Abstract

Near-infrared (NIR) fluorescent probes are attractive molecular tools for bioimaging because of their low autofluorescence interference, deep tissue penetration, and minimal damage to sample. However, most previously reported NIR probes exhibit small Stokes shift, typically less than 30 nm, and low fluorescence quantum yield, strictly limited contrast and spatial resolution for bioimaging. Herein, by expanding the π-conjugated system of rhodamine B, while, at the same time, keeping its rigid and planar structure, we reported an efficient NIR dye, **HN7**, with large stokes shift of 73 nm and fluorescence quantum yield as high as 0.72 in ethanol, values superior to those of such traditional cyanine NIR dyes as Cy5. Using **HN7**, living cells, tissues and mice were imaged, and the results showed significantly enhanced contrast, improved spatial resolution, and satisfactory tissue imaging depth when compared to Cy5. Moreover, the nonfluorescent spirocyclic structure of rhodamine B is an inherent component of **HN7**; therefore, our strategy provided a universal platform for the design of efficient NIR turn-on bioimaging probes for various targets. As a proof-of-concept, two different NIR probes, **HN7-N2** and **HN7-S** for NO and Hg^2+^, respectively, were designed, synthesized, and successfully applied for the imaging of NO and Hg^2+^ in living cells, tissues and mice, respectively, demonstrating the potential bioimaging applications of the new probes. In sum, this new type of dye may present new avenues for the development of efficient NIR fluorescent probes for contrast-enhanced imaging in biological applications.

## Abstract 2

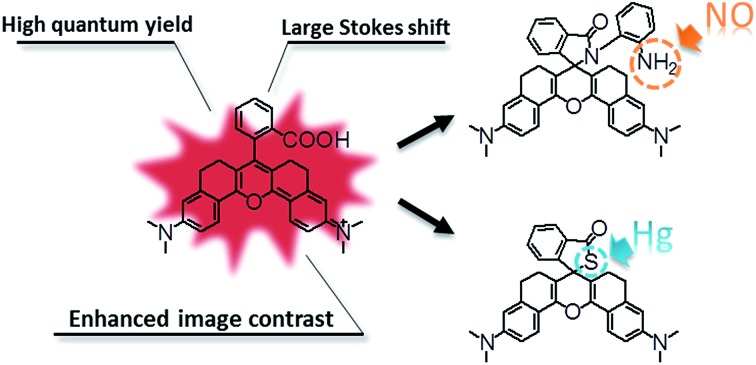
A universal sensing platform with significantly enhanced image contrast and apparent brightness in biological imaging applications.

## Introduction

The application of fluorescence imaging through staining with a fluorescent probe is characterized by high sensitivity, real-time spatial imaging and detection of targets in living cells or tissues with minimal damaging effects. As such, fluorescence imaging has become a facile and powerful tool for the detection of biologically relevant species with the ability to visualize morphological details and monitor various physiological processes in living systems.^1^–^8^ However, the imaging quality of this technique is strictly dependent on the photophysical properties of fluorescent dyes used for sample staining. It has recently been reported that NIR fluorescent imaging can minimize photodamage to biological samples, increase tissue penetration depth, and minimize interference from autofluorescence in living systems.^9^–^15^ Accordingly, many NIR fluorescent dyes have been developed in the past few years.^16^–^24^ However, most previously reported NIR fluorescent dyes suffer from serious self-quenching and excitation light backscattering as a result of their small Stokes shift, typically less than 30 nm, and most of them bear low fluorescence quantum yields, commonly much less than 0.1 in aqueous solution. Since these photophysical properties afford high background signal and low fluorescence signal for these dyes, low contrast for bioimaging can be expected. However, in order to obtain enhanced image contrast and optimal apparent brightness for bioimaging, the development of NIR dyes with both large stokes shift and high quantum yield would be the most efficient approach.

Cyanine dyes are classic NIR fluorophores with an odd number of carbons in a conjugated polymethine framework between two nitrogen-containing heterocycles.^25^ The maximal emission wavelengths of pentamethine cyanines (Cy5) and heptamethine cyanines (Cy7) reach well into the NIR region (>650 nm). However, based on their flexible molecular structure, traditional cyanine dyes suffer from several disadvantages, such as small Stokes shift, limited fluorescence quantum yield, poor photostability and undesirable aggregation.^26^ Moreover, cyanine dyes exhibit relatively high-lying occupied molecular orbital (HOMO) energy levels,^27^,^28^ making it difficult to develop cyanine-based fluorescent turn-on probes *via* photoinduced electron transfer (PET).^29^–^32^ These features limit the application of cyanine dyes in the construction of NIR probes for bioimaging.

The classic rhodamines possess excellent photophysical properties, such as high molar extinction coefficients, high fluorescence quantum yields, typically larger than 0.5,^33^ and excellent photostability.^33^–^36^ Moreover, the robust rhodamine spirolactam platform is perfect for constructing probes based on target-triggered fluorescence off–on switching, which affords large signal-to-background ratio (SBR).^34^–^36^ However, because of the limited π-conjugated system of xanthene derivatives, classic rhodamines, such as rhodamine 123, rhodamine 6G, and rhodamine B, only have emission wavelengths in the visible region (<600 nm), thus limiting their application in the bioimaging of living systems. In addition, based on the small Stokes shift of less than 30 nm with excitation back-scattering effect, rhodamine-based probes usually exhibit serious self-quenching and, hence, the tendency to report false-positive signals. Thus, to expand the application of the robust rhodamine spirolactam platform with enhanced image contrast and SBR in bioimaging, we considered developing functional rhodamine analogues emitting in the NIR region and possessing large Stokes shift and high quantum yield.

Therefore, in this work, we have expanded the π-conjugated system of rhodamine B beyond that of traditional rhodamine dyes, while keeping its rigid and planar structure, and developed a new series of far-red to NIR dyes named **HN1–7**. Among these dyes, the rigid and planar dye **HN7** exhibits excellent photophysical properties, such as fluorescence emission located in the NIR region (>670 nm), large Stokes shift (73 nm in PBS buffer), high fluorescence quantum yield (0.72 in EtOH; 0.25 in PBS buffer), excellent stability and aqueous solubility. To understand the structure–optical properties of **HN7**, we carried out quantum chemical calculations with the B3LYP exchange functional employing 6-31G* basis sets using a suite of Gaussian 09 programs.^37^ To study biological applicability, the new dye was tested for its performance in bioimaging. **HN7** was found to display an image contrast of 85-fold in living cells (10 μM) and 42-fold in living mice (5 nmol), values much greater than those of traditional rhodamine B and Cy5. The tissue penetration capability of **HN7** was slightly superior to that of Cy5. It is also worth noting that **HN7** showed obviously higher brightness and enhanced contrast over that of Cy5. Moreover, **HN7** possesses a rhodamine-like spirolactam structure, which provides a universal platform for the design of NIR turn-on bioimaging probes. To demonstrate the feasibility of this probe design, two different NIR probes, **HN7-N2** and **HN7-S** for NO and Hg^2+^, respectively, were designed, synthesized, and applied for imaging of NO and Hg^2+^ in living cells, tissues and mice.

## Results and discussion

### Design and synthesis of **HN** dyes

**Scheme 1:**
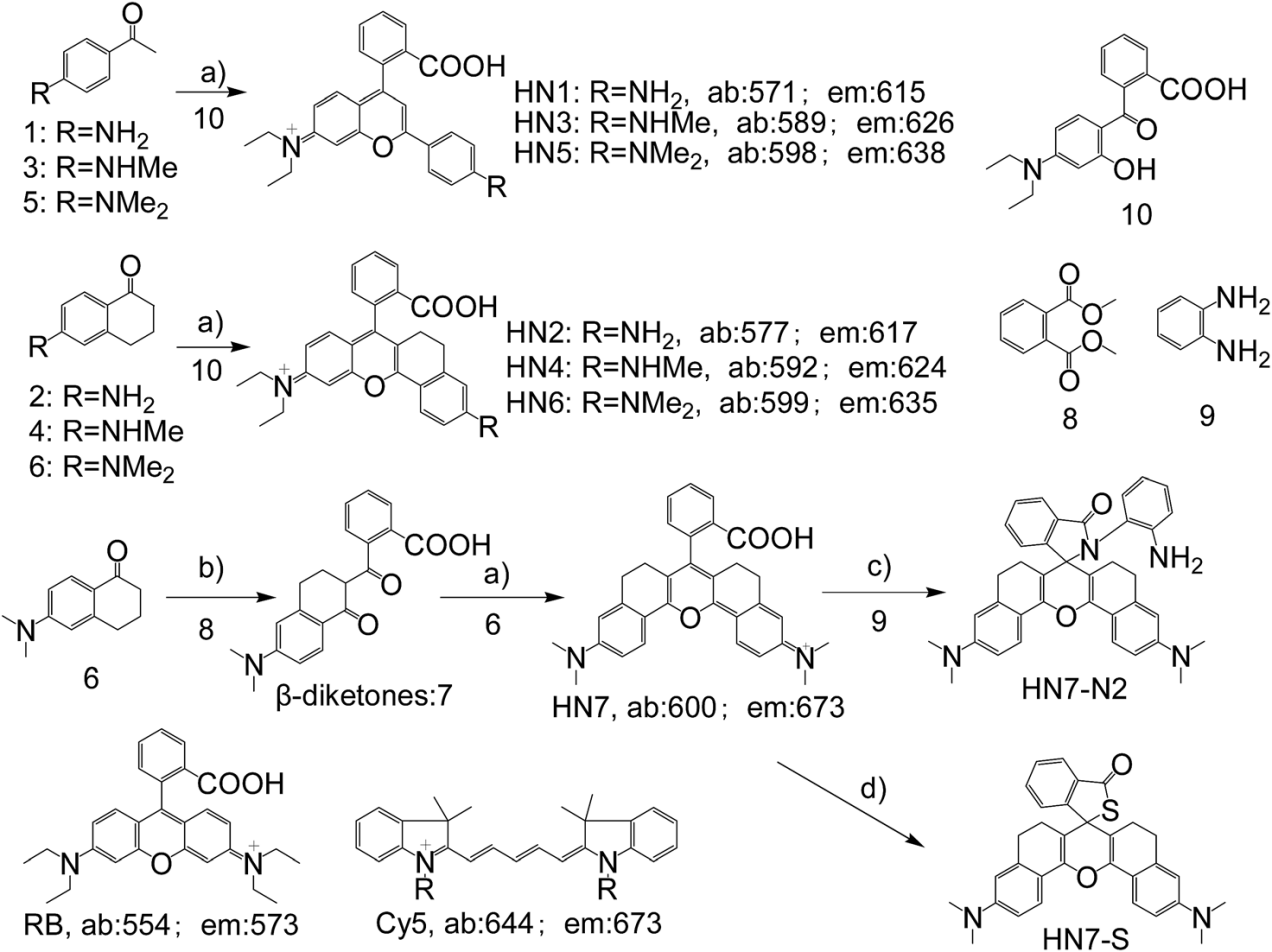
Synthesis of **HN1–7**, **HN7-N2** and **HN7-S**; structures of classical rhodamine B and Cy5. Conditions: (a) H_2_SO_4_, *Δ*, then 70% HClO_4_; (b) NaH, *Δ*; (c) DCC/DMAP or POCl_3_/TEA; (d) Lawesson's reagent.

In general, the emission wavelengths of organic dyes are highly dependent on intramolecular π-conjugated systems and substituent electronic effects, while rigid and planar molecular structures afford high fluorescence quantum yield for dyes. To test this hypothesis, several compounds possessing expanded π-conjugated systems and, thus, potential emission of red-shift fluorescence in comparison with traditional rhodamine dyes, were designed and synthesized. Compounds with a flexible structure, including **HN1**, **HN3**, and **HN5** with various amino substituents, were first synthesized (Scheme 1), using 4-aminoacetophenone derivatives as starting materials. By utilizing *N*-substituent 6-amino-3,4-dihydro-1(2*H*)-naphthalenone instead of 4-aminoacetophenone derivative as the starting reactant, we also designed and easily synthesized rigid dyes **HN2**, **HN4**, and **HN6** with an alkyl-ring to limit molecular rotation and vibration (Scheme 1).^38^ In order to achieve even more improved fluorescence properties, rigid dye **HN7** with a π-conjugated system expanded over that of **HN1–6** was further designed and synthesized by employing a β-diketone compound 7 as an intermediate, which was achieved by Claisen condensation between 6-amino-3,4-dihydro-1(2*H*)-naphthalenone and dimethyl phthalate.^39^ The structures of dyes **HN1–7** were characterized by ^1^H NMR, ^13^C NMR and MS (ESI).

### Optical properties

**Fig. 1:**
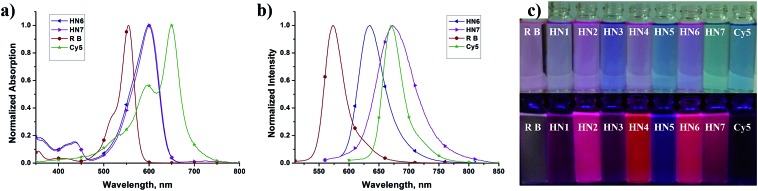
Absorption (a) and emission (b) spectra of **HN6** (navy-blue triangle), **HN7** (purple triangle) in pH 7.4 PBS buffer. The spectra of rhodamine B (wine hexagon) and Cy5 (olive-green star) are also shown for comparison. (c) Color and fluorescence images of rhodamine B, **HN1–7**, and Cy5 in pH 7.4 PBS buffer (3.0 μM, containing 10% EtOH).

We first tested the optical properties of **HN1–7**, rhodamine B and Cy5 in various solvents, including buffered aqueous solution (pH 7.4 PBS, containing 10% EtOH), EtOH, CH_2_Cl_2_, DMSO, and CH_3_CN. The absorption and emission profiles are illustrated in Fig. 1 and S1–5,† and the corresponding photophysical data are listed in Table 1 and S1–4.† In buffered aqueous solution, **HN1–7** display broad and intense maximum absorption bands in the 571–600 nm region, which is assigned to the 0–0 band of the S0 → S1 transition. Broad and weak absorption bands were found around 420 nm, which could be attributed to the S0 → S3 transition. Notably, the absorption of **HN7** exhibited an obvious blue-shift in comparison to Cy5, resulting in a larger Stokes shift in spite of its π-conjugated system which was expanded over that of rhodamine. On the other hand, the molar extinction coefficients (*ε*_max_) of all our new dyes are in the range of 120 000 M^–1^ cm^–1^ and thus superior to the classic rhodamines.^33^–^36^ Therefore, as shown in Fig. 1c (top), the rhodamine B solution displays a pink color to the naked eye, while the colors of the **HN1–7** solutions are all much deeper than the classic rhodamine B by their long absorption wavelengths and large molar extinction coefficients.

**Table 1:** Photophysical properties of dyes **HN1–7**, rhodamine B and Cy5 in pH 7.4 PBS buffer (containing 10% EtOH)

Dye	*λ*_abs_ ^*a*^/nm	*λ*_em_ ^*b*^/nm	SS^*c*^	*ε*_max_/M^–1^ cm^–1^	*Φ*_f_	*ε*_max_ *Φ*_f_/M^–1^ cm^–1^	*τ*/ns
**HN1**	571	615	44	117 900	0.06^*d*^	7074	0.79
**HN2**	577	617	40	119 200	0.43^*d*^	51 256	0.87
**HN3**	589	626	37	125 300	0.05^*d*^	6265	0.80
**HN4**	592	624	32	122 100	0.41^*d*^	50 061	0.74
**HN5**	598	638	40	129 500	0.02^*d*^	2590	0.66
**HN6**	599	635	36	123 300	0.37^*d*^	45 621	0.71
**HN7**	600	673	73	137 300	0.25^*e*^	34 325	0.88
RB	554	573	19	106 000	0.31^*f*^	32 860	0.94
Cy5	649	670	21	250 000	0.03^*e*^	7500	0.87

^*a*^The maximal absorption of the dye.

^*b*^The maximal emission of the dye.

^*c*^Stokes shift.

^*d*^ *Φ*_f_ is the relative fluorescence quantum yield estimated by using cresyl violet as a standard: *Φ*_f_ = 0.56 in EtOH.^40^

^*e*^ *Φ*_f_ is the relative fluorescence quantum yield estimated by using sulfoindocyanine dye Cy5 as a standard: *Φ*_f_ = 0.20 in PBS.^41^

^*f*^ *Φ*_f_ is the relative fluorescence quantum yield estimated by using rhodamine B as a standard: *Φ*_f_ = 0.72 in MeOH.^40^

Since their π-conjugated systems are expanded beyond those of the classic rhodamines, **HN1–6** display maximum fluorescence emission in the far-red region (615–638 nm) in PBS buffered solution. Remarkably, **HN7** displays Cy5-like fluorescence emission with maximum at 673 nm in PBS buffer and covers a significant part of the NIR region. Furthermore, according to the absorption and emission data, the Stokes shift of **HN7** is 73 nm in neutral buffered solution, which is superior to that of the classic rhodamine, fluorescein, **BODIPY**®, and cyanine dyes, all typically less than 30 nm. Such large Stokes shift could reduce background fluorescence for **HN7** and avoid self-quenching and false-positive signals from excitation backscattering effect.

Classic rhodamine dyes possess high fluorescence quantum yields, typically larger than 0.5, as a result of their rigid and planar molecular structures. As expected, compounds **HN1**, **HN3**, **HN5** with their flexible structure showed fluorescence quantum yields less than 0.22 in EtOH and less than 0.06 in aqueous solution.^40^ On the other hand, **HN2**, **HN4**, **HN6**, and **HN7** all possess rigid and planar molecular structures similar to rhodamine dyes, but they exhibit higher fluorescence quantum yields than the classic cyanine NIR dyes (*e.g.*, Cy5: *Φ*_f_ = 0.03 in buffered aqueous solution), ranging from 0.72 to 0.93 in EtOH and from 0.25 to 0.43 in aqueous solution.^41^ To evaluate a newly designed biological imaging reagent, brightness (*ε*_max_*Φ*_f_) is another important parameter to test, as it is involves imaging contrast and spatial resolution. Accordingly, the brightness of **HN7** was calculated to reach 34 325 M^–1^ cm^–1^ in buffered aqueous solution, which is superior to that of traditional rhodamine B and Cy5. All these results illustrate that our NIR dye, **HN7**, is suitable for bioimaging applications with enhanced brightness and contrast.

**Fig. 2:**
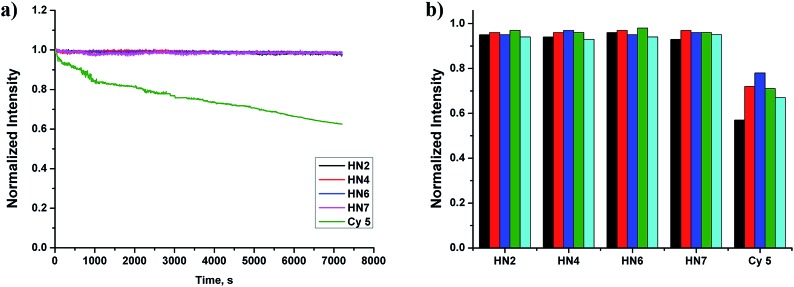
(a) Photostability of **HN2** (black), **HN4** (red), **HN6** (blue) and **HN7** (magenta) in pH 7.4 PBS buffer (containing 10% EtOH). The samples were continuously irradiated by a Xe lamp (150 W) at 5 nm slit width at the maximal absorption wavelength of **HN2**, **HN4**, **HN6**, and **HN7**. (b) Chemical stability of **HN2**, **HN4**, **HN6**, and **HN7** in pH 7.4 PBS buffer (containing 10% EtOH). The samples were reacted with various ROS (100 μM) for 120 min (black: HOCl, red: H_2_O_2_, blue: TBHP, olive-green: HO·, cyan: *t*BuO·).

Still another important parameter is photostability. By using continuous irradiation, as described above, we investigated the photostability of **HN2**, **HN4**, **HN6**, **HN7** and Cy5 in PBS buffer. Exactly as anticipated and as shown in Fig. 2a, over 98% of the initial fluorescence intensity of our **HN** dyes was retained after 120 min irradiation, while only 62% of Cy5 was retained, indicating that our rigid **HN** dyes have sufficient photostability for prolonged biological imaging. On the other hand, it is well known that many organic NIR dyes are easily bleached by reactive oxygen species (ROS).^42^ To evaluate the chemical stability of **HN** dyes under these conditions, the fluorescence profiles of **HN2**, **HN4**, **HN6**, **HN7** and Cy5 in the presence of various ROS (HOCl, H_2_O_2_, TBHP, HO·, *t*BuO·) were also investigated. As shown in Fig. 2b, more than 93% of the initial fluorescence intensity was retained after treatment of **HN** dyes with ROS in excess of 120 min, values much improved of those of Cy5. All these data suggest that our **HN** dyes have excellent photostability and the chemical stability required to resist photobleaching and common ROS in biological imaging applications.

**Fig. 3:**
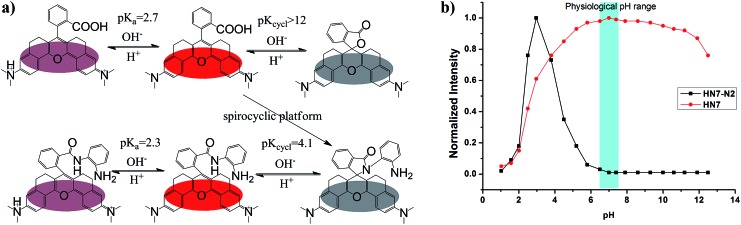
(a) The proposed structures and the putative transformations of **HN7** and **HN7-N2** at various pH values. (b) pH-dependence of the normalized fluorescence intensity of **HN7** (red circle) and **HN7-N2** (black square).

The pH value around the rhodamine analogues and their spirolactam derivatives typically affects performance because of protonation reaction in acidic conditions or cyclization reaction in alkaline conditions. Therefore, to study the practical applicability of the newly designed **HN** dyes, the effects of pH on their fluorescence response and a representative spirolactam derivative were investigated. As shown in Fig. 3, stable and high fluorescence were observed for pH values ranging from 5.0 to 11.0 for free **HN7**. However, fluorescence did decrease from protonation reaction in strong acidic conditions or the development of nonfluorescent spirocyclization in alkaline conditions, with p*K*_a_ at 2.7 and p*K*_cycl_ reaching 12.0. Moreover, the spirolactam derivative **HN7-N2** showed p*K*_cycl_ of about 4.1 and predominately existed in the nonfluorescent spirocyclic form with a broad range of pH values over 6.0. Therefore, just like the classic rhodamines and their spiro-ring derivatives,^33^–^36^ the fluorescence on–off switching of **HN** dyes and their spiro-ring derivatives would seem to have unlimited potential applications based on the robust rhodamine sensing platform.

### Density functional theory (DFT) studies

**Fig. 4:**
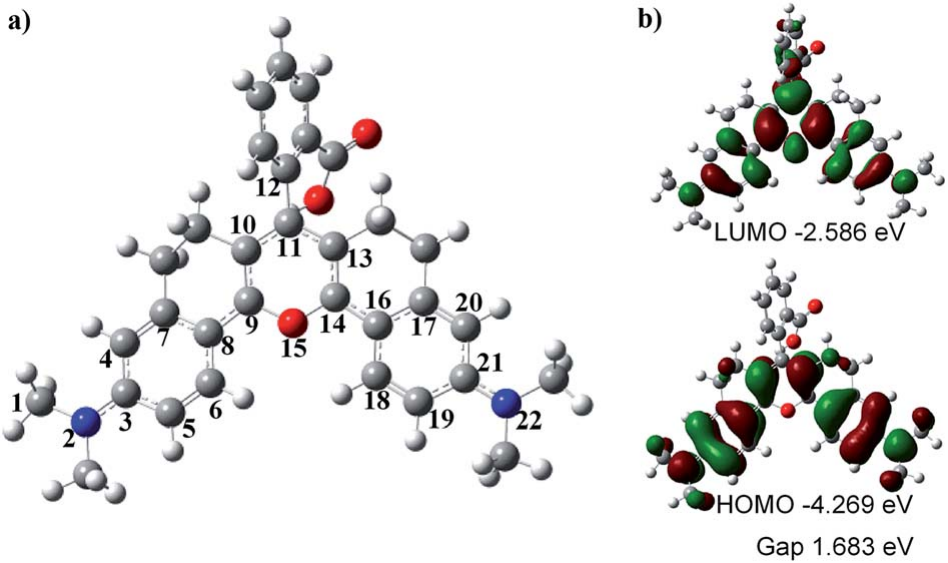
(a) DFT optimized structure of **HN7**. In the ball-and-stick representation, carbon, nitrogen, oxygen, and hydrogen atoms are colored in gray, blue, red, and white, respectively. (b) Molecular orbital plots (LUMO and HOMO) and HOMO/LUMO energy gaps of **HN7**.

To understand the optical properties of **HN7**, we carried out DFT calculations with the B3LYP exchange functional employing 6-31G* basis sets using a suite of Gaussian 09 programs.^37^Fig. 4 shows the representative optimized structure and the HOMO/LUMO molecular orbital plots of **HN7**. The C–C (N or O) bond lengths (in pm) of **HN7**, as determined by DFT calculations, are displayed in Table 2. As shown in Table 2, between the typical C–C single (154 pm) and double (134 pm) bonds, all C–C bond lengths of the conjugated fluorophore core are around 140 pm, which can be attributed to strong electronic delocalization and partial decrease in carbon–carbon bond length alternation (BLA, difference between consecutive single and double bonds) along the π-conjugated system. Such phenomenon can easily be proved by investigating the terminal C–N and C–O bond lengths in the conjugated system. For example, according to the calculated result based on C–N as a single bond, the bond length of C1–N2 is 147.1 pm, which is close to that of the typical C–N single bond (147 pm). Meanwhile, the bond lengths of N2–C3 and C21–N22 are 136.9 and 136.8 pm, respectively, which are intermediates between the lengths of C–N bond, as a single bond calculated above, and C=N, a double bond around 128 pm. Obviously, these bond length calculations support the strong electronic delocalization along the π-conjugated system of **HN7**.

**Table 2:** Representative X–X (C, N or O) bond lengths (pm) of **HN7** as determined by DFT calculations

X–X bond	Bond lengths (pm)	X–X bond	Bond lengths (pm)	X–X bond	Bond lengths (pm)
C1–N2	147.1	C8–C9	143.0	C14–C16	142.8
N2–C3	136.9	C9–C10	140.0	C16–C17	142.8
C3–C4	142.7	C10–C11	141.9	C16–C18	141.7
C3–C5	142.9	C11–C12	150.0	C18–C19	137.9
C5–C6	138.0	C11–C13	141.4	C19–C21	143.0
C6–C8	141.6	C13–C14	140.0	C20–C21	142.7
C8–C7	142.6	C14–O15	138.3	C20–C17	138.3
C4–C7	138.4	C9–O15	138.1	C21–N22	136.8

Notably, Fig. 4a shows that the benzoic acid moiety is highly twisted and approximately vertical to the fluorophore core with a dihedral angle of 78.7°, whereas the planar angle of the fluorophore core is confirmed to be 175°. Meanwhile, the C11–C12 length is 150.0 pm (Table 2), which is close to the typical C–C single bond (154 pm). All these data demonstrate that the benzoic acid moiety and the fluorophore core are effectively electronically decoupled. Thus, the absence of electronic conjugation between benzoic acid moiety and fluorophore core agrees very well with the finding that benzoic acid moiety makes no contribution to the HOMO/LUMO of **HN7** (see Fig. 4). But interestingly, the benzoic acid moiety carves up some electron density of the LUMO molecular orbital plots of **HN7**, which will greatly stabilize the LUMO and reduce the energy bandgap of the fluorophore core. In other words, this may interpret the large Stokes shift of **HN7**. Furthermore, the fluorophore core could maintain its planar configuration with a planar angle of 175°, thus leading to such superior optical properties as high fluorescence quantum yield. Taken together, the quantum chemical calculations fit well with the optical performance of **HN7**.

### Biological imaging studies

Our new NIR **HN7** dye possesses several excellent photophysical properties favorable for bioimaging applications. First, NIR fluorescence emission is always more suitable for biological imaging by its avoidance of autofluorescence effects of biological matrix. Second, the large Stokes shift could resit potentially serious self-quenching and false-positive signals from excitation backscattering. Third, its high fluorescence quantum yield should be appropriate for biological imaging with enhanced brightness and contrast. Therefore, to test these properties, **HN7** was applied for bioimaging in living systems.

**Fig. 5:**
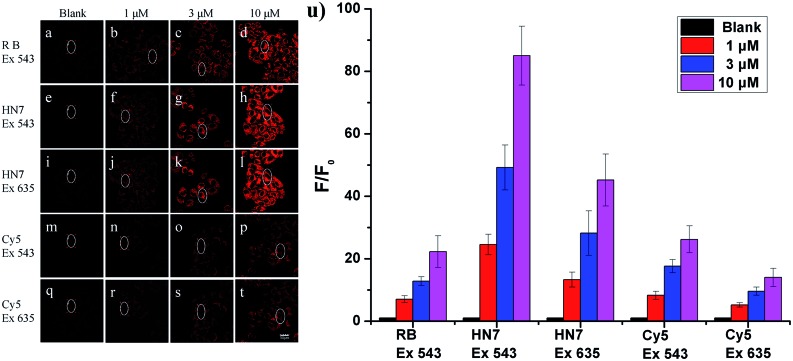
Confocal fluorescence images of HeLa cells incubated with various dyes at 37 °C for 60 min (from left to right: 0, 1, 3, 10 μM): (a–d) excitation at 543 nm for red emission of rhodamine B (560–630 nm); (e–h) excitation at 543 nm for red emission of **HN7** (650–720 nm); (i–l) excitation at 635 nm for red emission of **HN7** (650–720 nm); (m–p) excitation at 543 nm for red emission of Cy5 (650–720 nm); (q–t) excitation at 635 nm for red emission of **HN7** (650–720 nm). Scale bar = 30 μm. (u) Relative fluorescence intensity obtained from the selected ellipse region of (a–t), respectively, averaged and plotted as a ratio to blank (a, e, i, m, q).

To evaluate the imaging performance of **HN7**, it was first utilized in living cells, with rhodamine B and Cy5 chosen as reference compounds. Selectable confocal microscopy lasers were limited to 405, 458, 488, 515, 543, 635 nm excitation; therefore, by selecting excitation wavelength 543 nm for rhodamine B, **HN7** and Cy5, some exciting light energy was unavoidably lost. It should be noted that 635 nm was also selected as the excitation wavelength to fit the absorption of Cy5 for comparison. Meanwhile, the emission windows for rhodamine B (560–630 nm) and **HN7**, Cy5 (650–720 nm) were also confirmed. As a proof-of-concept, HeLa cells were incubated with **HN7**, rhodamine B and Cy5 (four groups: 0, 1, 3, and 10 μM, respectively) at 37 °C for 60 min, and the fluorescent imaging results were shown in Fig. 5a–t. Obviously, all the applied fluorescent dyes proved to be membrane permeable, emitting their fluorescence in the cytoplasm, rather than nuclear regions. Furthermore, using the same parameters, different levels of imaging brightness and contrast were observed for these compounds at various concentrations. Relative fluorescence intensity data were shown in Fig. 5u. First, weak fluorescence belonging to either cellular autofluorescence or excitation light backscattering could be observed in Fig. 5a, where it was particularly serious, as well as Fig. 5i and q.^43^ Thus, rhodamine B showed satisfactory brightness, even at the low concentration of 1 μM. However, the imaging contrast, as determined by the relative fluorescence intensity enhancement ratios to blank, was critically limited by 7-, 12.8-, and 22.3-fold. In contrast, because of its large Stokes shift and high fluorescence quantum yield, under two different excitation wavelengths of 543 nm and 635 nm, **HN7** not only showed brightness comparable to that of rhodamine B, but also considerable relative fluorescence intensity enhancement ratios as high as 85-fold, with a high enhancement ratio of 24.6-fold, even at the low concentration of 1 μM. While the relative fluorescence intensity enhancement ratios of Cy5 were tolerable, imaging brightness was very weak and not suitable for visual observation. Therefore, compared to classic rhodamine B and Cy5, our new **HN7** bore excellent imaging properties, including NIR emission, high brightness and enhanced contrast. As such, these results demonstrate the potential *in vivo* imaging applications of **HN7**, especially for trace fluorescence imaging.

The cytotoxicity of **HN7** in HeLa cells was also examined using the MTS (3-(4,5-dimethylthiazol-2-yl)-5-(3-carboxymethoxyphenyl)-2-(4-sulfophenyl)-2*H*-tetrazolium) assay. Fig. S5† showed that **HN** dyes exhibited low cytotoxicity after incubation in HeLa cells for 48 h. Such desirable property may also render the new **HN** dyes suitable for applications in living systems.

**Fig. 6:**
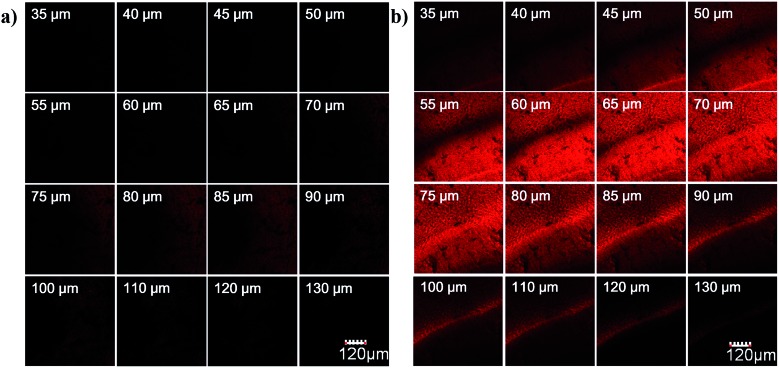
Depth fluorescence images of Cy5 ((a), 5 μM) and **HN7** ((b), 5 μM) in tissues. The changes of fluorescence intensity with scan depth were determined by spectral confocal microscopy (Olympus, FV1000) in the *z*-scan mode (from 0 to 150 μm; step size: 1 μm). The images were collected at 650–720 nm (red channel). Scale bar = 120 μm.

To evaluate the imaging depth of the new NIR dye, **HN7** was further applied for tissue imaging of rat liver frozen slices, and the changes of fluorescence signal intensity with scan depth were then determined by confocal microscopy (Olympus, FV1000) in *z*-scan mode. As shown in Fig. 6, experimental results showed that **HN7** possessed satisfactory tissue imaging capability at depths of 40–120 μm. Meanwhile, Cy5 was capable of tissue imaging at depths of 75–100 μm, indicating that the tissue penetration capability of **HN7** was slightly superior to that of Cy5. It is worth noting that **HN7** showed obviously enhanced brightness and contrast when compared to Cy5 for imaging frozen rat liver tissue slices. These data demonstrated the potential of **HN7** in tissue imaging applications.

**Fig. 7:**
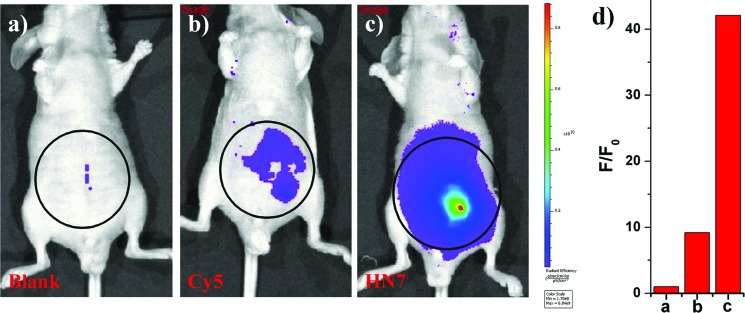
Fluorescent images of mice (pseudocolor). Thirty minutes after dye injection, mice were imaged using a Caliper VIS Lumina XR small animal optical *in vivo* imaging system with an excitation filter 605 nm and a Cy5.5 emission filter. (a) The blank group; (b) the Cy5-incubated group; (c) the **HN7**-incubated group; (d) quantification of fluorescence emission intensity from the selected circle region of (a–c) was averaged and plotted as a ratio to blank.

NIR light causes only minimal photodamage to samples and has the advantage of deep tissue penetration and reduced interference from autofluorescence in living animals; therefore, NIR dyes are more favorable for *in vivo* imaging. Compared to the traditional NIR dye Cy5, our unique **HN7** dye exhibits a similar fluorescence emission spectrum, but has additional advantages, such as larger Stokes shift, higher fluorescence quantum yield, greater photostability and satisfactory aqueous solubility, all of which could provide low background, high fluorescence signal and enhanced contrast for bioimaging applications in living animals. As confirmation, we further tested the analytical characteristics of Cy5 and **HN7***in vivo*. Fluorescence images of four-week-old male BALB/c nude mice were first taken with two well-separated wavelengths of an excitation filter 605 nm and a Cy5.5 emission filter. Significantly, as shown in Fig. 7a, without any intraperitoneal injection, the blank image showed extremely weak endogenous fluorescence intensity, which could be attributed to weak biological autofluorescence minimal excitation backscattering effect. Next, by intraperitoneal injection with **HN7** and Cy5 (5 nmol), compared to the blank group, a considerable enhanced fluorescence intensity of 42-fold was observed for the **HN7**-incubated group (Fig. 7c), whereas fluorescence enhancement of the Cy5-incubated group compared to the blank group was estimated to be only 9.2-fold by its low fluorescence quantum yield (Fig. 7b). These results demonstrate that this novel **HN7** dye is superior to the traditional NIR Cy5 dye for bioimaging applications in living animals.

### Application of the **HN** dye for design of fluorescent probes

**Scheme 2:**
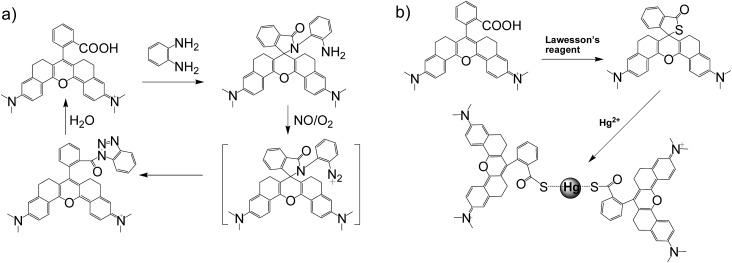
(a) NO-induced ring opening of **HN7-N2**. (b) Hg^2+^-induced ring opening of **HN7-S**.

Apart from its excellent photophysical properties, **HN7** also possesses a rhodamine-like spirolactam structure, which provides a universal platform for the design of efficient NIR turn-on bioimaging probes for various targets. To demonstrate the feasibility of this probe design, two different NIR probes, **HN7-N2** and **HN7-S**, were designed and synthesized for sensing NO and Hg^2+^ (Scheme 2), by utilizing a phenylenediamine unit for sensing NO^44^ and a thiolactone for sensing Hg^2+^,^45^,^46^ respectively. NO and Hg^2+^ were chosen as model targets. NO has been discovered to play a critical role in the cardiovascular system, the central and peripheral nervous systems and the immune systems. It has also been recognized as an important molecule in several physiological and pathological processes.^47^,^48^ Hg^2+^ is one of the most toxic heavy metal ions, as demonstrated by even a small amount of Hg^2+^ ion which can cause permanent deterioration of brain, heart, kidney, stomach, intestine, central nervous and endocrine systems.^49^,^50^ Therefore, considerable attention could be devoted to our new fluorescent probes for the detection of NO and Hg^2+^ with sufficient selectivity, sensitivity and imaging performance.

**Fig. 8:**
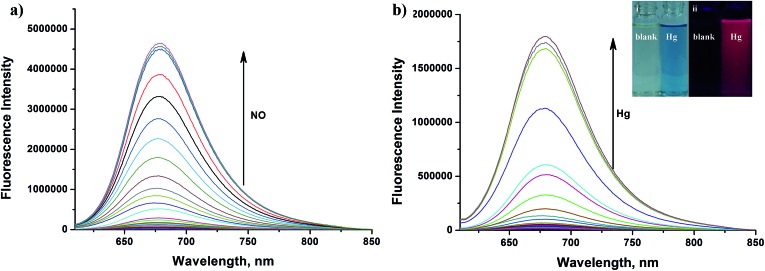
(a) Fluorescence spectra of **HN7-N2** (5 μM) in the presence of various concentrations of NO (from diethylamine NONOate sodium salt, 0–100 μM) in PBS buffer (0.01 M, pH 7.4, containing 10% EtOH) with excitation at 600 nm. Inset: change in color (i) and fluorescence (ii) in the absence of NO (left) and in the presence of 100 μM NO (right). (b) Fluorescence spectra of **HN7-S** (5 μM) in the presence of various concentrations of Hg^2+^ (0–10 μM) in PBS buffer (0.01 M, pH 7.4, containing 10% EtOH) with excitation at 600 nm. Inset: change in color (i) and fluorescence (ii) in the absence of Hg^2+^ (left) and in the presence of 10 μM Hg^2+^ (right).

As shown in Fig. 8a, similar to classic rhodamine spirocyclic derivatives, the **HN7-N2** free probe in buffered aqueous solution was nearly colorless and fluorescence-inactive, indicating predominance of the spirocyclic form. However, with the introduction of NO (from diethylamine NONOate sodium salt), **HN7-N2** instantly exhibited blue color, as determined by the naked eye, accompanied by considerable red fluorescence (Fig. 8a inset). A significant turn-on absorption (Fig. S6†) and fluorescence response (Fig. 8a) was observed following the NO-induced ring-opening reaction of **HN7-N2** over other biologically relevant species (Fig. S7†). Interestingly, upon the addition of 20 equiv. of NO, a 1500-fold fluorescence enhancement at 676 nm was observed. Fig. S8† shows the calibration curve of the **HN7-N2** probe, and it exhibits a linear range from 20 nM to 1 μM, with a detection limit of 8 nM (3*s*/slope). Similar results were also obtained when using the **HN7-S** probe for Hg^2+^ detection (Fig. 8b and S10–12†). Obviously, our analytical data *in vitro* is as well as the reported rhodamine-based probes (Tables S5–S6†).^44^–^46^ Thus, these results indicate that **HN7** could be employed as a universal platform to design efficient NIR bioimaging probes for various analytes.

**Fig. 9:**
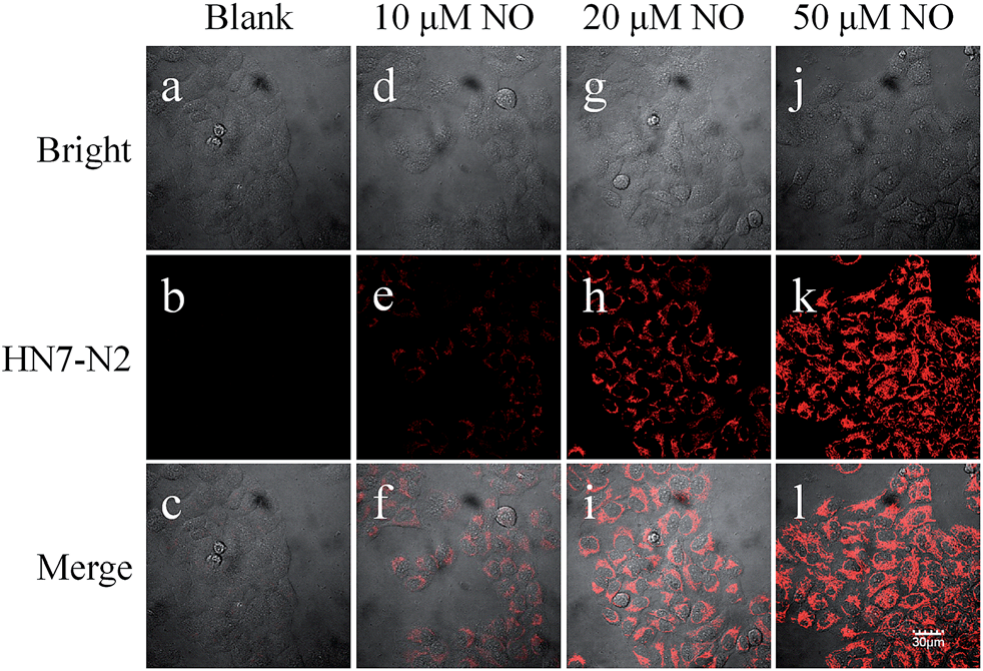
Confocal fluorescence imaging of NO in HeLa cells with **HN7-N2** (10 μM) for 30 min and then NO donor, diethylamine NONOate (from left to right: 0, 10, 20, 50 μM, respectively) for 30 min. Excitation at 543 nm for red emission of **HN7-N2** (650–720 nm).

The excellent photophysical properties of **HN7** make both **HN7-N2** and **HN7-S** probes especially favorable for bioimaging applications. To demonstrate the practical applicability of the probes in biological systems, we chose **HN-N2** as the model probe and carried out fluorescence imaging experiments in living cells, tissue slices and mice. First, intracellular NO was monitored using confocal fluorescence imaging. In Fig. 9, HeLa cells incubated with **HN7-N2** (10 μM) for 30 min showed no observable fluorescence signal, indicating that the **HN7-N2** probe maintained its spirocyclic form in cells. When HeLa cells preincubated with **HN7-N2** were further treated with different concentrations of NO donor, diethylamine NONOate (10, 20, 50 μM), a dramatic increase in fluorescence brightness was observed, suggesting that the new probe was cell permeable and could be used as an imaging tool to quantify the concentration of NO in living cells.

**Fig. 10:**
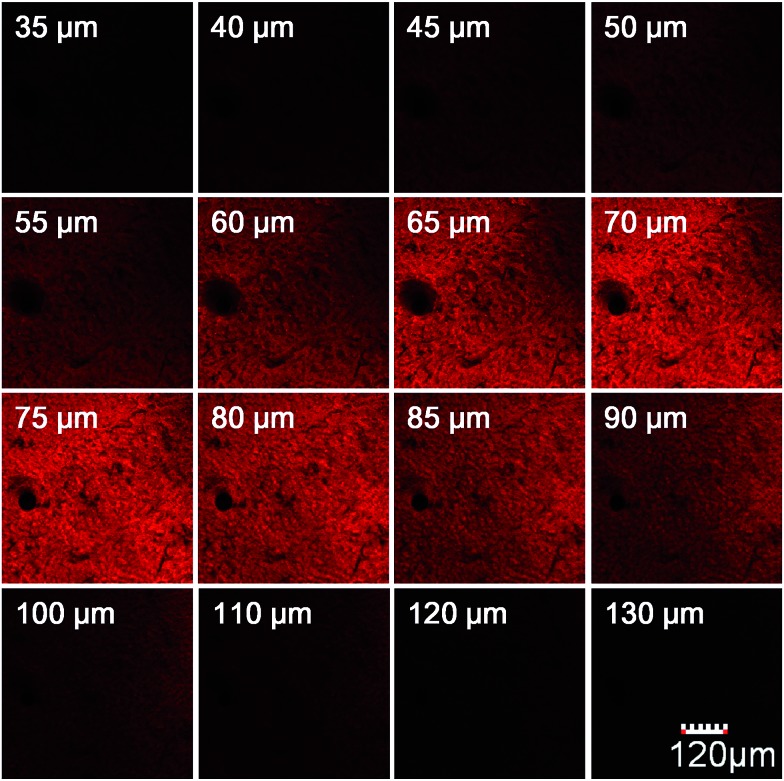
Depth fluorescence images of 10 μM **HN7-N2** for NO detection in tissues were obtained with spectral confocal microscopy (Olympus, FV1000). The changes of fluorescence intensity with scan depth were determined by spectral confocal microscopy (Olympus, FV1000) in the *z*-scan mode (from 0 to 150 μm; step size: 1 μm). The images were collected at 650–720 nm (red channel). The slice was cultured with **HN7-N2** (5 μM) for 60 min and then NO (50 μM) for another 60 min.

**Fig. 11:**
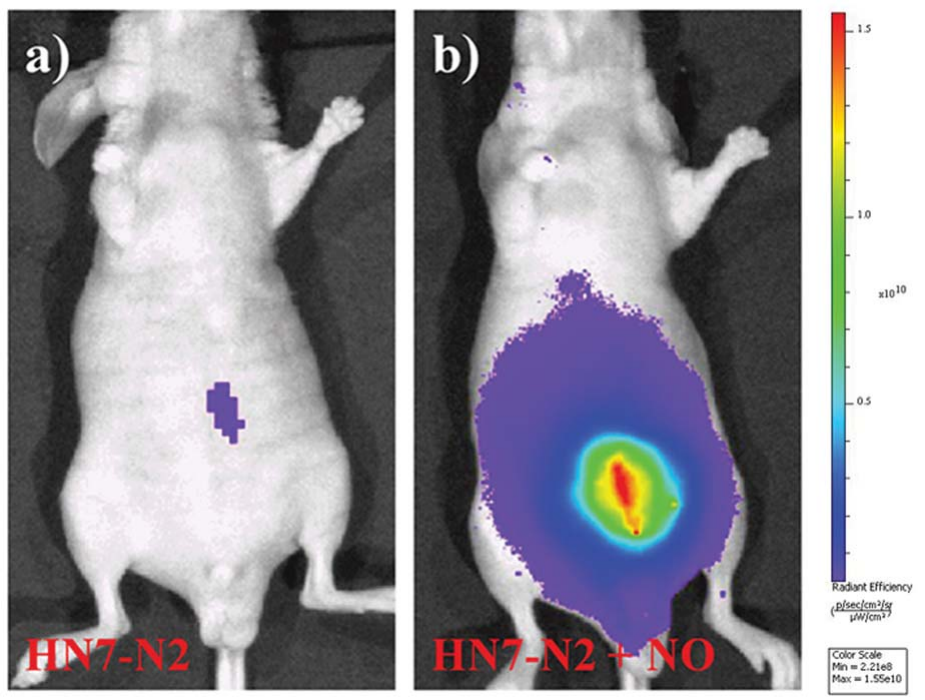
Fluorescent images of mice (pseudocolor). The mice were imaged using a Caliper VIS Lumina XR small animal optical *in vivo* imaging system with an excitation filter 605 nm and a Cy5.5 emission filter. Left: imaging of control group after intraperitoneal injection of **HN7-N2** probe (20 nanomoles) for 30 min; right: intraperitoneal injection of **HN7-N2** probe (20 nanomoles) for 30 min, followed by intraperitoneal injection of NO donor (50 nanomoles). The mice were imaged 30 min after NO donor injection.

We further used **HN7-N2** for NO imaging in tissue of rat liver frozen slice. The changes of fluorescence intensity with scan depth were determined by confocal microscopy (Olympus, FV1000) in the *z*-scan mode. As shown in Fig. 10, in the presence of NO, the **HN7-N2** probe was capable of tissue imaging at depths of 45–110 μm. These data showed that the **HN7** probe had good tissue penetration and staining ability. **HN7-N2** was also used for NO detection *in vivo*. For four-week-old male BALB/c nude mice, the control group was only given an intraperitoneal injection of **HN7-N2** (20 nmol). The experimental group was first given an intraperitoneal injection of **HN7-N2** probe (20 nmol), followed by an intraperitoneal injection with NO donor, diethylamine NONOate (50 nmol), after 30 min. The mice were then imaged using a Caliper VIS Lumina XR small animal optical in *vivo* imaging system with an excitation filter 605 nm and a Cy5.5 emission filter. As shown in Fig. 11, the control group showed weak fluorescence, indicating that the probe maintained the fluorescence-inactive spirolactam form *in vivo*. However, mice treated with both **HN7-N2** and NO exhibited significantly stronger fluorescence intensity than the control group, indicating that **HN7-N2** could be applied for imaging NO *in vivo*.

Fluorescence imaging experiments of **HN7-S** for Hg^2+^ in living cells, tissue slices and mice were also performed, and results were similar to those of **HN7-N2** for NO were obtained (Fig. S13–15†). All these results indicated that **HN7** is an efficient and universal platform for the design of NIR bioimaging probes for various analytes with enhanced brightness and contrast.

## Conclusions

In summary, by introducing a practical strategy of constructing a rigid molecular structure, we have successfully developed a novel NIR functional fluorescent dye, **HN7**, with excellent photophysical properties, such as large Stokes shift and high fluorescence quantum yield, which could afford significantly enhanced image contrast and apparent brightness in biological imaging applications when compared to traditional rhodamine B and Cy5. Furthermore, the spirocyclic structure of rhodamine B is inherent in **HN7**; as such, our strategy provided a universal platform for the design of efficient NIR fluorescent turn-on bioimaging probes for various targets. As a proof-of-concept, two different NIR probes, **HN7-N2** and **HN7-S** for NO and Hg^2+^, were designed, synthesized, and successfully applied for the imaging of NO and Hg^2+^ in living cells, tissues and mice, respectively, demonstrating the potential bioimaging applications of the new probes. Thus, the new type of dye presented herein may open up new avenues for the development of efficient NIR fluorescent probes for contrast-enhanced biological imaging applications.

## Supplementary Material

Supplementary information
