# Supplementary material for: A unique approach toward near-infrared fluorescent probes for bioimaging with remarkably enhanced contrast
Source: Chem Sci. 2016 Jan 4;7(3):2275–85. doi: 10.1039/c5sc04014k (PMC5977507; doi:10.1039/c5sc04014k)
Supplement: Supplementary file 1 [file SC-007-C5SC04014K-s001.pdf]

Electronic Supplementary Material (ESI) for Chemical Science.
This journal is © The Royal Society of Chemistry 2016

Supporting information for

A Unique Approach toward Near-Infrared Fluorescent Probes for Bioimaging with Remarkably Enhanced Contrast

Yi-Jun Gong,[a] Xiao-Bing Zhang,*[b] Guo-Jiang Mao,[a] Li Su,[a] Hong-Min Meng,[a] Weihong Tan,[b] Suling Feng, [a] and Guisheng Zhang,*[a]

[a] Collaborative Innovation Center of Henan Province for Green Manufacturing of Fine Chemicals; Key Laboratory of Green Chemical Media and Reactions, Ministry of Education; School of Chemistry and Chemical Engineering, Henan Normal University, Xinxiang, Henan 453007, P. R. China.

[b] State Key Laboratory for Chemo/Biosensing and Chemometrics, College of Chemistry and Chemical Engineering, Hunan University, Changsha 410082, China.

E-mail: zgs6668@yahoo.com, xbzhang@hnu.edu.cn

Table of Contents

Materials and instruments S4

Measurement of fluorescence quantum yields S4

Cell incubation and fluorescence imaging S5

In vitro cytotoxicity of **HN** dyes S5

Preparation and Staining of Rat Liver Tissue Slices S6

Fluorescence Imaging in Living Mice S6

Synthesis S7

Figure S1 .... S11

Table S1 .... S11

Figure S2 .... S12

Table S2 .... S12

Figure S3 .... S13

Table S3 .... S13

Figure S4 .... S14

Table S4 .... S14

Figure S5 .... S15

Figure S6 .... S15

Figure S7 .... S16

Figure S8 .... S16

Figure S9 .... S16

Figure S10 .... S17

Figure S11 .... S17

Figure S12 .... S18

Figure S13 .... S18

Figure S14 .... S19

Figure S15 .... S19

Figure S16 .... S20

Table S5 .... S21

Table S6 .... S21

NMR and MS spectra S22

References S28

Materials and instruments. All reagents were purchased from commercial suppliers and used without further purification. Solvents were purified by standard methods. All solutions were prepared using ultrapure water, which was prepared through a Millipore Milli-Q water purification system (Billerica, MA, USA). TLC analysis was performed on silica gel plates, and column chromatography was conducted over silica gel (mesh 200-300), both of which were obtained from Qingdao Ocean Chemicals. ^{1}H and ^{13}C NMR spectra were recorded on a Bruker DRX-400 spectrometer operating at 400 and 100 MHz with chemical shifts reported as ppm (in DMSO-d6, TMS as internal standard). Mass spectrometric data LC-MS analyses were performed using an Agilent 1100 HPLC/MSD spectrometer. The pH measurements were carried out on a Mettler-Toledo Delta 320 pH meter. UV-vis absorption spectra were recorded with a Shimadzu UV-2450 spectrophotometer. All fluorescence intensity measurements were carried out on a Fluoromax-4 spectrofluorometer (HORIBA JobinYvon, Edison, NJ). Fluorescence lifetime measurements were carried out on a FLS-980 fluorescent spectrophotometer (Edinburgh Instrument, UK). CellTiter 96® Aqueous One Solution Cell Proliferation Assay (MTS) was purchased from Promega. Fluorescence images of cells were obtained using an Olympus FV1000 laser scanning confocal microscope (Japan). Fluorescence images of mice were carried out on a Caliper VIS Lumina XR small animal optical *in vivo* imaging system.

Measurement of fluorescence quantum yields.[1,2] Fluorescence quantum yields of **HN1-7**, rhodamine B and Cy5 were determined by using cresyl violet ($\Phi_f = 0.56$ in EtOH), sulfoindocyanine dye Cy5 ($\Phi_f = 0.20$ in PBS) and rhodamine B ($\Phi_f = 0.72$ in MeOH) as fluorescence standards. The quantum yield was calculated using the following equation:

$$\Phi_{F(X)} = \Phi_{F(S)} \left(A_S F_X / A_X F_S\right) \left(n_X / n_S\right)^2$$

where Φ_F is the fluorescence quantum yield, A is the absorbance at the excitation wavelength, F is the area under the corrected emission curve, and n is the refractive index of the solvents used. Subscripts s and x refer to the standard and to the unknown, respectively. The solutions of compounds **HN1-7**, rhodamine B, Cy5 and standards were kept absorptions below 0.05.

Cell incubation and fluorescence imaging. Living HeLa cells were obtained from the Biomedical Engineering Center of Hunan University (Changsha, China) and cultured in DMEM (Dulbecco's modified Eagle's medium) supplemented with 10% FBS (fetal bovine serum) at 37 ºC in a 5% CO_2 atmosphere.

For comparative experiments, the living HeLa cells were incubated with **HN7**, rhodamine B and Cy5 (all 0, 1, 3 and 10 μM, respectively) for 60 min. For detection experiments, the living HeLa cells were incubated with probe (**HN7-N2** or **HN7-S**) for 30 min as the control group, and the HeLa cells pretreated with probe were further incubated with analyte (NO or Hg^{2+}) for another 30 min. All cells were washed three times with PBS, and the fluorescence images were then performed through an Olympus FV1000-IX81 confocal fluorescence microscope with excitation wavelength at 543 nm or 635 nm, emission wavelength 560-600 nm for rhodamine B, and emission wavelength 650-720 nm for **HN7**, **HN7-N2**, **HN7-S** and Cy5. Scale bar = 30 μm.

***In vitro* cytotoxicity of HN dyes.** Cytotoxicity study was performed using the CellTiter 96 Aqueous One Solution cell proliferation assay ((3-(4,5-dimethylthiazol-2-yl)-5- (3-carboxymethoxyphenyl)-2-(4-sulfophenyl)-2H-tetrazolium) (MTS) (Promega, USA)). HeLa cells (2 x 10^5) were seeded in a 30-mm glass bottomed dish and incubated for 48 h. After removing cell medium, cells were first incubated with **HN7** (1, 2, 5, and 10 μM) in 1 mL Dulbecco's Phosphate-Buffered Saline (D-PBS, 1.47 mM KH_2PO_4, 8.06 mM Na_2HPO_4, 137.93 mM NaCl, 2.67 mM KCl) at 37 ºC for 2 h to allow sufficient uptake. Afterwards, supernatant was removed from the test well following centrifugation, and 200 μL of fresh cell culture medium were added. After another 48 h of incubation, a 6 x diluted MTS solution (120 μL/well) in DMEM medium solution was added to each well and incubated at 37 ºC for 2 h. The absorbance value at 490 nm was determined by a microplate reader.

Preparation and Staining of Rat Liver Tissue Slices. Tissue slices were prepared from rat liver frozen slices. A side of the tissue was cut flat using a vibrating-blade microtome. The slices were cultured with 5 μM **HN7**, 5 μM Cy5, 10 μM **HN7-N2** and then 100 μM NO or 10 μM **HN7-S** and then 20 μM Hg^{2+} in an incubator at 37 °C for 60 min, respectively, followed by washing three times with PBS before imaging. The images were collected in red emission 650-720 nm upon excitation at 635 nm. Scale bar = 120 μm.

Fluorescence Imaging in Living Mice: Four-week-old male BALB/c nude mice were obtained from SJA Co., Ltd. (Changsha, China). All animal operations were in accord with institutional animal use and care regulations, according to protocol No. SYXK (Xiang) 2008-0001, approved by Laboratory Animal Center of Hunan.

For comparative experiments, the blank group was anesthetized without any treatment. The second group was given an intraperitoneal injection of Cy5 (5 nanomoles). The third group was given an intraperitoneal injection of **HN7** (5 nanomoles).

For analyte detection *in viv*o, the control group was only given an intraperitoneal injection of probe (**HN7-N2** or **HN7-S**). The second group was given an intraperitoneal injection with probe, followed by intraperitoneal injection with analyte (NO or Hg^{2+}) after 30 min.

Before imaging, the mice were anesthetized by inhalation of 5% isoflurane in 100% oxygen. The animals were placed into the imaging chamber and kept under anesthetic using an isoflurane gas anesthesia system. The nude mice were imaged using a Caliper VIS Lumina XR small animal optical *in vivo* imaging system. Unless otherwise stated, imaging mode for all experiments was set as excitation scan, and Input/Em was chosen as 605 nm for excitation with Cy5.5 filter for emission channel. All fluorescence images were acquired with autoexposure (FOV, 10 cm; f/stop 2; bin, high resolution), and fluorescence intensity was scaled as units of photons per second per square centimeter per steradian ($ps^{-1}cm^{-2}sr^{-1}$).

Synthesis

Synthesis of HN1-6. 2-(4-diethylamino-2-hydroxybenzoyl)-benzoic acid (3 mmol) and N-substituent ketone (3 mmol) were added to concentrated H_2SO_4 (15 mL) at 0 °C. The reaction mixture was heated at 90 °C for 1.5 h, cooled down, and poured onto ice. Perchloric acid (70%, 1 mL) was then added. The resulting precipitate was filtered off and washed with cold water (100 mL), which was purified by silica gel flash chromatography using CH_2Cl_2 to CH_2Cl_2/ethanol (100:1 to 20:1) as eluent to afford **HN1-6** as a purple-green solid (yield > 80%).

HN1. ^{1}H NMR (DMSO-d6, 400 MHz) δ (ppm): 1.41 (t, 6H), 3.55 (q, 4H), 6.22 (d, J = 8.0 Hz, 1H), 6.45 (d, J = 9.2 Hz, 2H), 6.68 (d, J = 8.0 Hz, 1H), 6.92 (s, 1H), 7.02 (d, J = 9.2 Hz, 2H), 7.17 (s, 1H), 7.38 (t, J = 8.4 Hz, 1H), 7.55 (d, J = 8.4 Hz, 1H), 7.81 (d, J = 8.8 Hz, 1H), 8.23 (t, J = 8.8 Hz, 1H); ^{13}C NMR (DMSO-d6, 100 MHz) δ (ppm): 13.54, 48.52, 98.57, 103.98, 113.21, 115.26, 119.87, 121.13, 123.10, 126.02, 128.41, 130.75, 131.94, 133.85, 137.11, 137.98, 143.86, 147.22, 149.64, 152.29, 158.72, 165.28. MS (ESI) *m/z* 413.2. M^+.

HN2. ^{1}H NMR (DMSO-d6, 400 MHz) δ (ppm): 1.33 (t, 6H), 3.22 (t, 2H), 3.27 (t, 2H), 3.67 (q, 4H), 6.58 (s, 1H), 6.77 (d, J = 9.6 Hz, 1H), 6.92 (d, J = 8.4 Hz, 1H), 7.11 (d, J = 8.4 Hz, 1H), 7.22 (s, 1H), 7.39 (d, J = 9.6 Hz, 1H), 7.58 (t, J = 10.4 Hz, 1H), 7.88 (t, J = 9.2 Hz, 1H), 8.12 (d, J = 10.4 Hz, 1H), 8.23 (d, J = 9.2 Hz, 1H); ^{13}C NMR (DMSO-d6, 100 MHz) δ (ppm): 14.30, 26.75, 31.21, 52.10, 105.42, 109.23, 110.57, 115.24, 121.26, 122.78, 124.76, 125.41, 126.89, 128.63, 131.22, 132.68, 135.77, 136.57, 140.52, 147.69, 148.96, 153.45, 155.81, 167.21. MS (ESI) *m/z* 439.2. M^+.

HN3. ^{1}H NMR (DMSO-d6, 400 MHz) δ (ppm): 1.42 (t, 6H), 3.12 (s, 3H), 3.59 (q, 4H), 6.17 (d, J = 8.4 Hz, 1H), 6.39 (d, J = 8.4 Hz, 1H), 6.72 (d, J = 9.6 Hz, 2H), 6.82 (s, 1H), 6.97 (s, 1H), 7.15 (d, J = 9.6 Hz, 2H), 7.31 (d, J = 7.2 Hz, 1H), 7.72 (t, J = 7.6 Hz, 1H), 7.85 (d, J = 7.6 Hz, 1H), 8.17 (t, J = 7.2 Hz, 1H); ^{13}C NMR (DMSO-d6, 100 MHz) δ (ppm): 13.77, 29.31, 50.31, 99.12, 102.92, 112.74, 115.02, 118.65, 120.56, 123.01, 125.88, 127.59, 130.25, 131.77, 133.41, 136.82, 137.54, 141.14, 142.39, 148.17, 150.25, 157.18, 166.75. MS (ESI) *m/z* 427.4. M^+.

HN4. ^{1}H NMR (DMSO-d6, 400 MHz) δ (ppm): 1.37 (t, 6H), 3.05 (s, 3H), 3.28 (t, 2H), 3.35 (t, 2H), 3.77 (q, 4H), 6.32 (d, J = 8.8 Hz, 1H), 6.48 (s, 1H), 6.72 (d, J = 7.2 Hz, 1H), 6.88 (d, J = 7.2 Hz, 1H), 7.05 (s, 1H), 7.19 (d, J = 8.8 Hz, 1H), 7.38 (t, J = 7.6 Hz, 1H), 7.71 (t, J = 7.2 Hz, 1H), 8.02 (d, J = 7.2 Hz, 1H), 8.15 (d, J = 7.6 Hz, 1H); ^{13}C NMR (DMSO-d6, 100 MHz) δ (ppm): 13.89, 27.72,

30.24, 32.78, 53.32, 103.27, 108.55, 109.45, 113.85, 120.19, 122.23, 123.54, 125.13, 127.28, 128.23, 131.24, 133.12, 135.53, 138.21, 141.47, 146.56, 147.88, 151.42, 154.97, 166.77. MS (ESI) *m/z* 453.3. M^+.

HN5. ^{1}H NMR (DMSO-d6, 400 MHz) δ (ppm): 1.44 (t, 6H), 3.08 (s, 6H), 3.55 (q, 4H), 6.35 (d, *J* = 7.6 Hz, 1H), 6.65 (d, *J* = 7.6 Hz, 1H), 6.85 (d, *J* = 8.8 Hz, 2H), 6.98 (s, 1H), 7.15 (d, *J* = 8.8 Hz, 2H), 7.31 (m, 2H), 7.68 (s, 1H), 7.87 (d, *J* = 7.2 Hz, 1H), 7.98 (t, *J* = 7.2 Hz, 1H); ^{13}C NMR (DMSO-d6, 100 MHz) δ (ppm): 14.23, 35.72, 53.22, 97.15, 101.97, 113.12, 114.21, 118.22, 121.24, 122.33, 125.14, 126.32, 130.70, 133.29, 134.42, 136.14, 137.68, 138.82, 143.44, 145.28, 151.47, 159.28, 163.32. MS (ESI) *m/z* 441.3. M^+.

HN6. ^{1}H NMR (DMSO-d6, 400 MHz) δ (ppm): 1.20 (t, *J* = 6.8 Hz, 6H), 2.43 (m, 2H), 2.88 (m, 2H), 3.19 (s, 6H), 3.58 (q, *J* = 6.8 Hz, 4H), 6.75 (s, 1H), 6.87 (d, *J* = 9.6 Hz, 1H), 6.93 (d, *J* = 9.2 Hz, 1H), 7.10 (d, *J* = 9.6 Hz, 1H), 7.25 (s, 1H), 7.41 (d, *J* = 7.6 Hz, 1H), 7.78 (t, *J* = 7.6 Hz, 1H), 7.86 (t, *J* = 7.6 Hz, 1H), 8.19 (m, 2H); ^{13}C NMR (DMSO-d6, 100 MHz) δ (ppm): 14.64, 29.24, 32.56, 37.54, 55.01, 102.34, 106.22, 109.21, 112.71, 117.43, 120.03, 121.79, 125.22, 126.80, 128.21, 130.46, 133.41, 135.30, 140.89, 142.64, 147.41, 149.56, 153.33, 155.61, 165.84. MS (ESI) *m/z* 467.2. M^+.

Synthesis of HN7. Dimethyl phthalate (4 g, 20.6 mmol) and 6-dimethylamino-3, 4-dihydro-1(2H)-naphthalenone (378 mg, 2 mmol) were dissolved in dry THF (100 mL), and then NaH in PE (1 g) was added under an atmosphere of nitrogen. The mixture was stirred at 60 °C for 24 h and then poured into ice water. HCl (6 M) was added to neutralize excess base. The mixture was extracted with CH_2Cl_2 three times, and the organic phase was combined and dried over sodium sulfate. After removal of solvent, the yellow residue β-diketones, **HN7** dye intermediate was used directly without further purification. Then β-diketones, **HN7** dye intermediate and 6-dimethylamino-3,4-dihydro-1(2H)-naphthalenone (378 mg, 2 mmol) were added to concentrated H_2SO_4 (10 mL) at 0 °C. The reaction mixture was heated at 90 °C for 4 h, cooled down, and poured onto ice. Perchloric acid (70%, 1 mL) was then added, and the resulting precipitate was filtered off and washed with cold water (100 mL), which was purified by silica gel flash chromatography using CH_2Cl_2 to CH_2Cl_2/ethanol (200:1 to 20:1) as eluent to afford **HN7** as a green solid (yield: 27%). **HN7**. ^{1}H NMR (DMSO-d6, 400 MHz) δ (ppm): 2.83 (s, 12H), 3.16 (t, *J* = 9.6 Hz, 4H), 3.29 (t, *J* = 9.6 Hz, 4H), 6.97 (s, 2H), 7.01 (d, *J* = 9.2 Hz, 2H), 7.10 (d, *J* = 9.2 Hz, 2H), 7.47 (d, *J* = 7.2 Hz, 1H), 7.81 (t, *J* = 7.6

Hz, 1H), 7.88 (t, J = 7.6 Hz, 1H), 8.22 (d, J = 7.6 Hz, 1H); ^{13}C NMR (DMSO-d6, 100 MHz) δ (ppm): 27.13, 29.54, 52.48, 109.21, 113.51, 121.63, 123.82, 127.26, 129.60, 131.55, 135.64, 136.12, 144.52, 148.33, 150.62, 152.45, 158.74, 160.06, 168.20. MS (ESI) *m/z* 491.4. M^+.

Synthesis of HN7-N2. Route 1: To a CH_2Cl_2 solution (30 mL) of **HN7** (98 mg, 0.2 mmol), DCC (41 mg, 0.2 mmol) and DMAP (2.5 mg, 0.02 mmol) were added at room temperature in the dark, and the mixture was stirred for 0.5 h. After *o*-phenylenediamine (215 mg, 2 mmol) was added, the solution was stirred for 6 h. The solvent was removed under reduced pressure, and the residue was purified by silica gel flash column chromatography using petroleum ether/ethyl acetate (10:1 to 3:1) as eluent to afford **HN7-N2** as a pale yellow solid (yield: 53%).

Route 2: Phosphorus oxychloride (0.5 mL) was carefully added dropwise to a stirred solution of **HN7** (98 mg, 0.2 mmol) in 1,2-dichloroethane (20 mL) over 1 min, and the mixture was then refluxed for 4 h. After cooling down to room temperature, the solvent was removed under reduced pressure to provide **HN7** acyl chloride as glutinous oil. The residue was added to dry acetonitrile (10 mL) to form a solution to which a solution of *o*-phenylenediamine (435 mg, 4 mmol) and triethylamine (1 mL, 7.17 mmol) in dry acetonitrile (5 mL) was added dropwise. The mixture was stirred overnight at room temperature. After the solvent was removed under reduced pressure, the residue was redissolved in CH_2Cl_2 and washed with water (100 mL × 3). The organic phase was then dried over anhydrous sodium sulfate. The solvent was removed under reduced pressure, and the residue was purified by silica gel flash column chromatography using petroleum ether/ethyl acetate (10:1 to 3:1) as eluent to afford **HN7-N2** as a pale yellow solid (yield: 31%).

HN7-N2. ^{1}H NMR (DMSO-d6, 400 MHz) δ (ppm): 2.48 (t, J = 7.6 Hz, 4H), 2.71 (t, J = 7.6 Hz, 4H), 2.89 (s, 12H), 6.24 (s, 2H), 6.38 (d, J = 12.0 Hz, 2H), 6.50 (d, J = 12.0 Hz, 2H), 6.85 (d, J = 11.6 Hz, 1H), 6.99 (d, J = 6.8 Hz, 1H), 7.31 (t, J = 12.0 Hz, 1H), 7.39 (t, J = 12.0 Hz, 1H), 7.51 (m, 2H), 7.80 (d, J = 9.6 Hz, 1H), 8.19 (d, J = 11.6 Hz, 1H) ; ^{13}C NMR (DMSO-d6, 100 MHz) δ (ppm): 24.14, 26.17, 46.52, 70.28, 107.42, 111.31, 116.52, 117.25, 119.58, 120.98, 123.10, 123.88, 125.13, 126.22, 126.97, 128.36, 129.57, 133.54, 135.59, 141.95, 144.21, 148.67, 154.27, 159.40, 166.32. MS (ESI) *m/z* 580.3. M^+.

Synthesis of HN7-S. HN7 (98 mg, 0.2 mmol), Lawesson's reagent (90 g, 0.22 mmol), and toluene (20 mL) were mixed and heated to 90 °C. After stirring for 6 h, the solvent was removed under

reduced pressure. The residue was purified by silica gel flash column chromatography using petroleum ether/ethyl acetate (20:1 to 5:1) as eluent to afford **HN7-S** as a pale green solid (yield: 17%). MS (ESI) *m/z* 507.3. $[M+H]^+$.

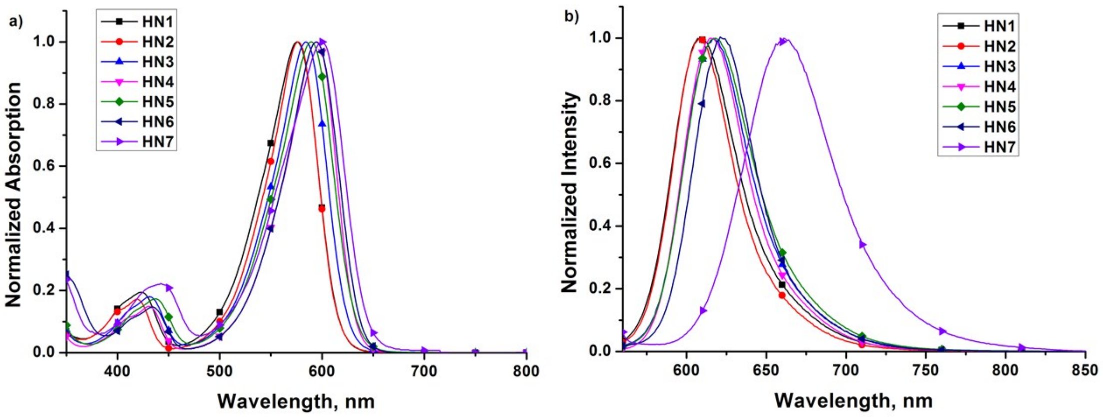

Figure S1. Absorption (a) and emission (b) spectra of **HN1** (black square), **HN2** (red circle), **HN3** (blue triangle), **HN4** (magenta-red triangle), **HN5** (olive-blue square), **HN6** (navy blue triangle), **HN7** (violet triangle) in EtOH.

Table S1. Photophysical properties of dyes **HN1-7** in EtOH.

dye	λ_{abs}/nm[a]	λ_{em}/nm[b]	SS[c]	ε_{max}/$M^{-1}cm^{-1}$	Φ_f	$\varepsilon_{max}\Phi_f$/$M^{-1}cm^{-1}$	τ/ns
HN1	575	609	34	125300	0.22[d]	27566	2.74
HN2	576	608	32	121600	0.93[d]	113088	2.65
HN3	584	617	33	132400	0.17[d]	22508	2.16
HN4	594	615	21	122100	0.87[d]	106227	2.95
HN5	589	618	29	131500	0.09[d]	11835	2.32
HN6	594	622	28	121500	0.85[d]	103275	2.88
HN7	599	661	62	130400	0.72[e]	93888	2.70

[a]The maximal absorption of the dye. [b]The maximal emission of the dye. [c]Stokes shift. [d]Φ_f is the relative fluorescence quantum yield estimated by using cresyl violet as a standard: $\Phi_f = 0.56$ in EtOH. [e]Φ_f is the relative fluorescence quantum yield estimated by using sulfoindocyanine dye Cy5 as a standard: $\Phi_f = 0.20$ in PBS.

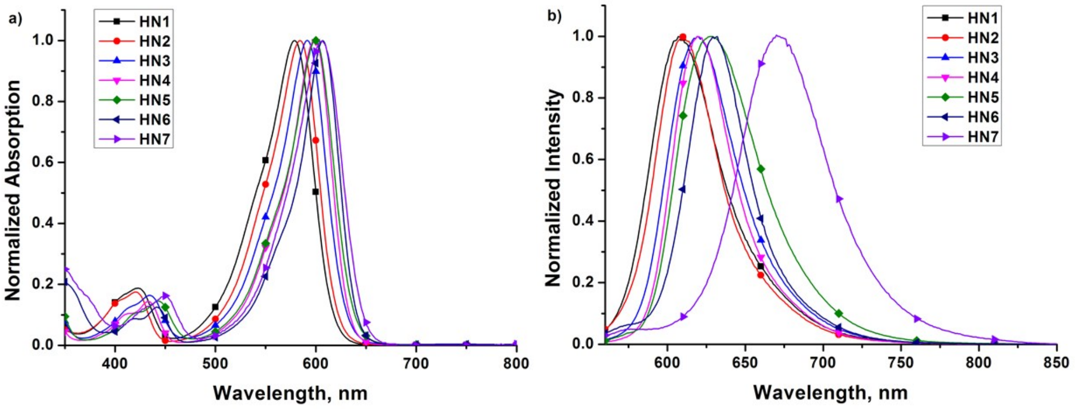

Figure S2. Absorption (a) and emission (b) spectra of **HN1** (black square), **HN2** (red circle), **HN3** (blue triangle), **HN4** (magenta-red triangle), **HN5** (olive-blue square), **HN6** (navy blue triangle), **HN7** (violet triangle) in CH_2Cl_2.

Table S2. Photophysical properties of dyes **HN1-7** in CH_2Cl_2.

dye	λ_{abs}/nm[a]	λ_{em}/nm[b]	SS[c]	$\varepsilon_{max}/M^{-1}cm^{-1}$	Φ_f	$\varepsilon_{max}\Phi_f/M^{-1}cm^{-1}$	τ/ns
HN1	579	607	28	127800	0.23[d]	29394	5.13
HN2	584	611	27	123000	0.95[d]	116850	5.41
HN3	591	619	28	131600	0.17[d]	22372	4.87
HN4	599	620	21	122400	0.90[d]	110160	5.32
HN5	599	629	30	133800	0.11[d]	14718	5.04
HN6	607	632	25	127500	0.85[d]	108375	4.74
HN7	606	672	66	137400	0.73[e]	100302	5.68

[a]The maximal absorption of the dye. [b]The maximal emission of the dye. [c]Stokes shift. [d]Φ_f is the relative fluorescence quantum yield estimated by using cresyl violet as a standard: $\Phi_f = 0.56$ in EtOH. [e]Φ_f is the relative fluorescence quantum yield estimated by using sulfoindocyanine dye Cy5 as a standard: $\Phi_f = 0.20$ in PBS.

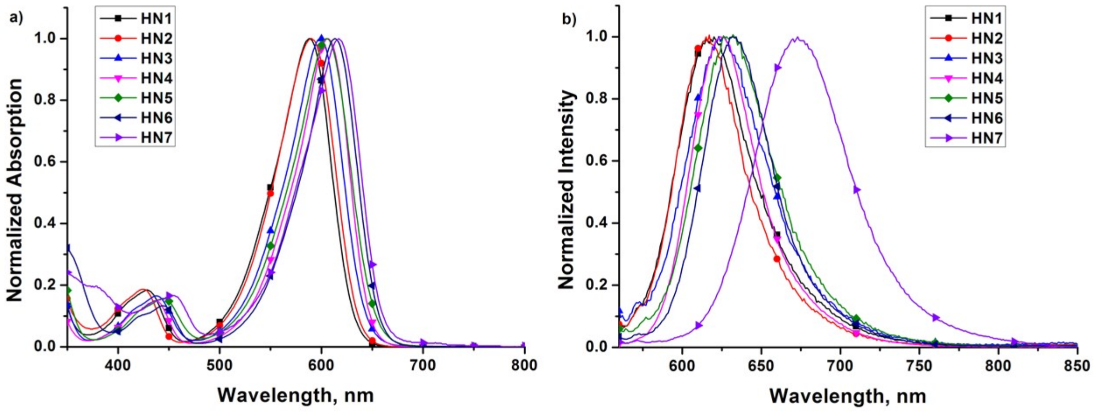

Figure S3. Absorption (a) and emission (b) spectra of **HN1** (black square), **HN2** (red circle), **HN3** (blue triangle), **HN4** (magenta-red triangle), **HN5** (olive-blue square), **HN6** (navy blue triangle), **HN7** (violet triangle) in DMSO.

Table S3. Photophysical properties of dyes **HN1-7** in DMSO.

dye	λ_{abs}/nm[a]	λ_{em}/nm[b]	SS[c]	ε_{max}/$M^{-1}cm^{-1}$	Φ_f	$\varepsilon_{max}\Phi_f$/$M^{-1}cm^{-1}$	τ/ns
HN1	588	620	32	117900	0.16[d]	18864	2.14
HN2	590	615	25	109200	0.88[d]	96096	2.50
HN3	599	626	27	118800	0.15[d]	17820	1.97
HN4	606	624	18	111700	0.86[d]	96062	2.05
HN5	605	628	23	122300	0.12[d]	14676	2.01
HN6	613	632	19	112000	0.80[d]	89600	2.22
HN7	617	673	56	124200	0.67[e]	83214	2.47

[a]The maximal absorption of the dye. [b]The maximal emission of the dye. [c]Stokes shift. [d]Φ_f is the relative fluorescence quantum yield estimated by using cresyl violet as a standard: $\Phi_f = 0.56$ in EtOH. [e]Φ_f is the relative fluorescence quantum yield estimated by using sulfoindocyanine dye Cy5 as a standard: $\Phi_f = 0.20$ in PBS.

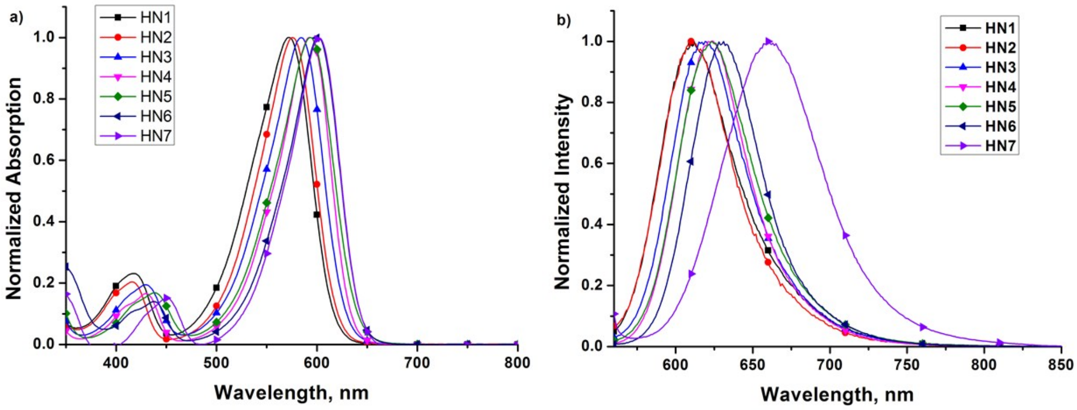

Figure S4. Absorption (a) and emission (b) spectra of **HN1** (black square), **HN2** (red circle), **HN3** (blue triangle), **HN4** (magenta-red triangle), **HN5** (olive-blue square), **HN6** (navy blue triangle), **HN7** (violet triangle) in CH_3CN.

Table S4. Photophysical properties of dyes **HN1-7** in CH_3CN.

dye	λ_{abs}/nm[a]	λ_{em}/nm[b]	SS[c]	ε_{max}/$M^{-1}cm^{-1}$	Φ_f	$\varepsilon_{max}\Phi_f$/$M^{-1}cm^{-1}$	τ/ns
HN1	571	612	41	128800	0.19[d]	24472	3.11
HN2	575	610	35	119400	0.90[d]	107460	3.15
HN3	584	619	35	125800	0.17[d]	21386	2.94
HN4	592	623	31	114200	0.87[d]	99354	3.30
HN5	593	623	30	129800	0.12[d]	15576	3.08
HN6	601	631	30	112300	0.82[d]	92086	3.24
HN7	602	660	58	121500	0.71[e]	86265	3.06

[a]The maximal absorption of the dye. [b]The maximal emission of the dye. [c]Stokes shift. [d]Φ_f is the relative fluorescence quantum yield estimated by using cresyl violet as a standard: $\Phi_f = 0.56$ in EtOH. [e]Φ_f is the relative fluorescence quantum yield estimated by using sulfoindocyanine dye Cy5 as a standard: $\Phi_f = 0.20$ in PBS.

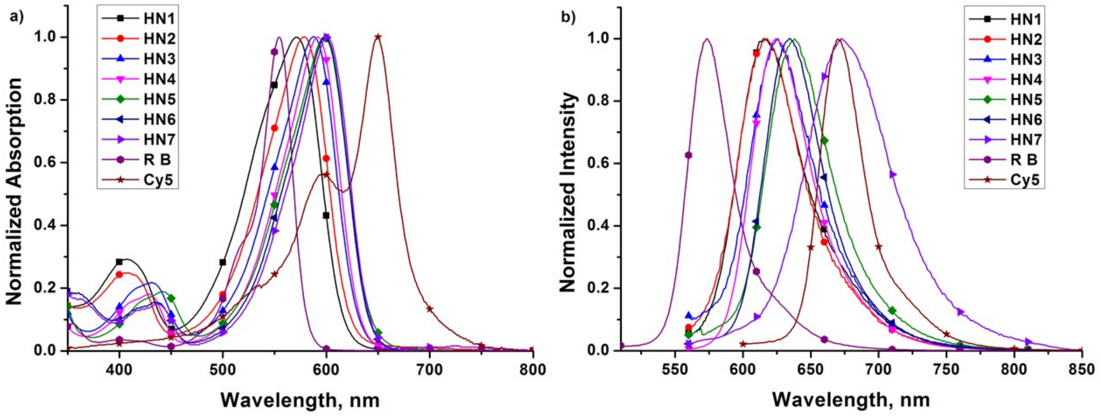

Figure S5. Absorption (a) and emission (b) spectra of **HN1** (black square), **HN2** (red circle), **HN3** (blue triangle), **HN4** (magenta-red triangle), **HN5** (olive-blue square), **HN6** (navy-blue triangle), **HN7** (violet triangle) in pH 7.4 PBS buffer. The spectra of rhodamine B (purple circle) and Cy5 (wine star) are also shown for comparison.

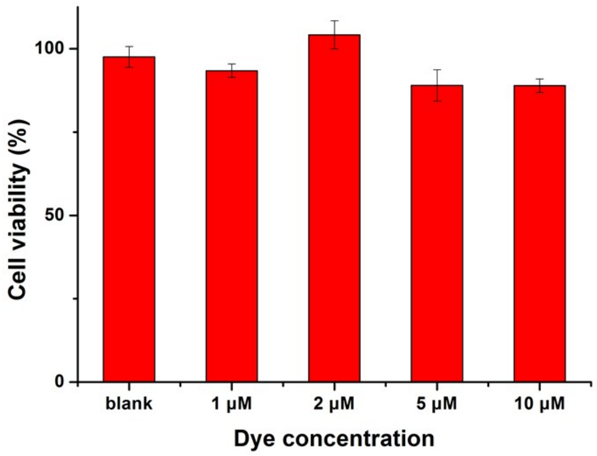

Figure S6. Cytotoxicity of **HN7** in HeLa cells. **HN7** was incubated in HeLa cells for 48 h.

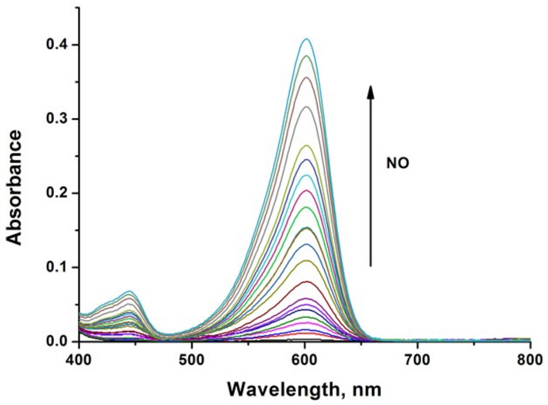

Figure S7. The UV-vis of **HN7-N2** (5 μM) in the presence of various concentrations of NO (0-10 μM) in PBS buffer (0.01 M, pH 7.4, containing 10% EtOH).

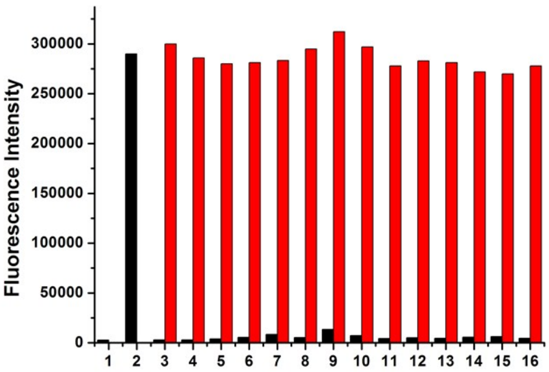

Figure S8. Fluorescence response of **HN7-N2** (5 μM) to 1 μM of NO or 10 μM of other various biologically relevant species (the black bar portion) and to the mixture of 1 μM of NO (the red bar portion) with 10 μM of other various biologically relevant species: 1, blank; 2, NO; 3, Cys; 4, GSH; 5, His; 6, NO_2^-; 7, NO_3^-; 8, ascorbic acid; 9, $ONOO^-$; 10, HNO; 11, HO·; 12, TBHP; 13, *t*BuO·; 14, H_2O_2; 15, HClO; 16, $O_2^{\cdot -}$.

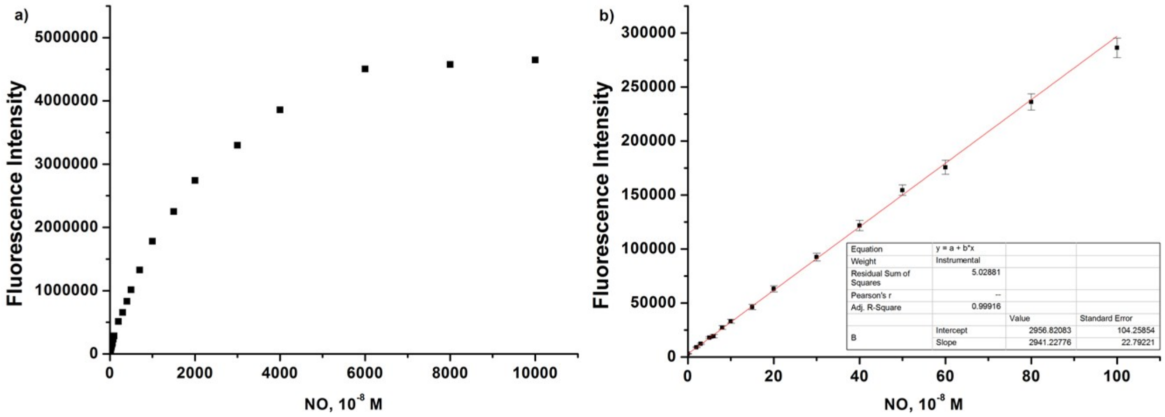

Figure S9. (a) Fluorescence intensity of **HN7-N2** (5 μM) at 676 nm *vs*. NO concentration (0-100 μM). (b) Calibration curve of the **HN7-N2** probe (5 μM) at 676 nm. The curve was plotted with the fluorescence intensity *vs*. NO concentration (0-1 μM).

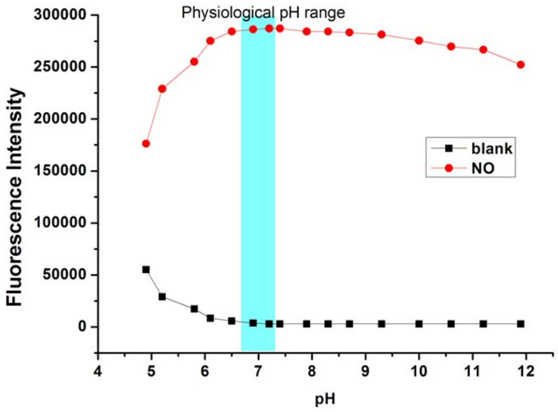

Figure S10. Effect of pH on the fluorescence intensity at 676 nm of free **HN7-N2** (5 μM, black square) and after addition of 1 μM NO (red circle).

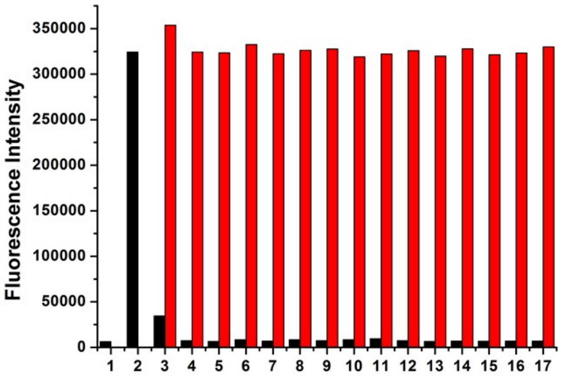

Figure S11. Fluorescence response of **HN7-S** (5 μM) to 0.5 μM of Hg^{2+} or 5 μM of other various metal ions (the black bar portion) and to the mixture of 0.5 μM of Hg^{2+} (the red bar portion) with 5 μM of other various metal ions (except K^+ and Na^+): 1, blank; 2, Hg^{2+}; 3, Ag^+; 4, Cd^{2+}; 5, Cr^{3+}; 6, Cu^{2+}; 7, Fe^{3+}; 8, Fe^{2+}; 9, Ca^{2+}; 10, Co^{2+}; 11, Ni^{2+}; 12, Mn^{2+}; 13, Fe^{2+}; 14, Pd^{2+}; 15, Mg^{2+}; 16, Zn^{2+}, 17, Pb^{2+}.

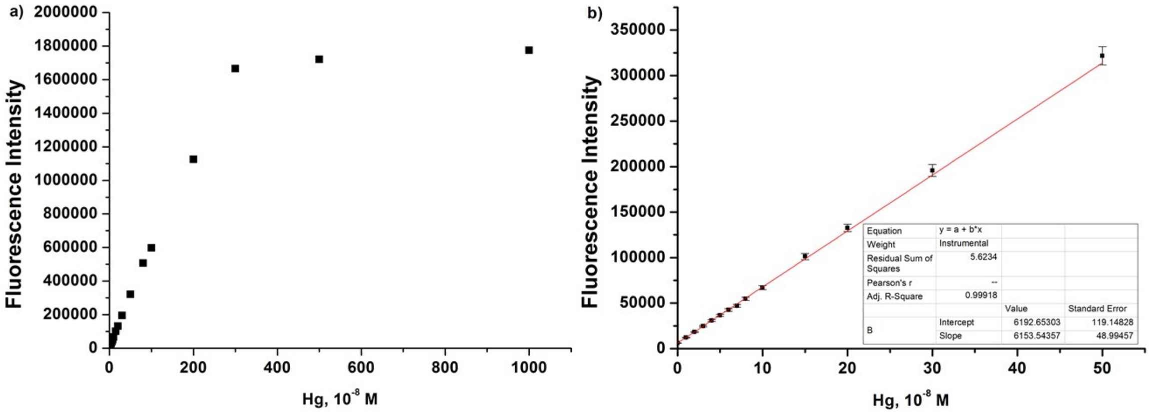

Figure S12. (a) Fluorescence intensity of **HN7-S** (5 μM) at 678 nm *vs*. Hg concentration (0-10 μM). (b) Calibration curve of the **HN7-S** probe (5 μM) at 678 nm. The curve was plotted with the fluorescence intensity *vs*. Hg concentration (0-0.5 μM).

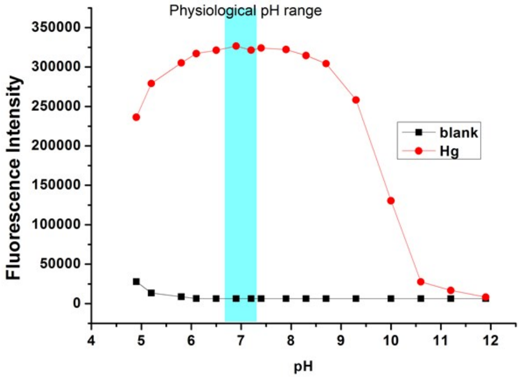

Figure S13. Effect of pH on the fluorescence intensity at 678 nm of free **HN7-S** (5 μM, black square) and after addition of 0.5 μM Hg^{2+} (red circle).

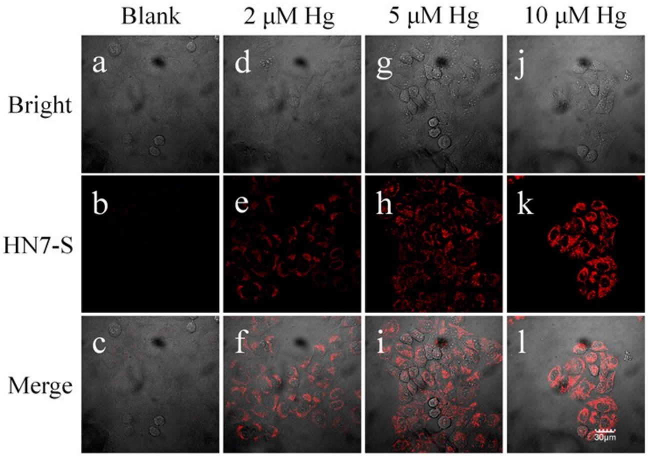

Figure S14. Confocal fluorescence imaging of Hg^{2+} in HeLa cells with **HN7-S** (10 μM) for 30 min and then Hg^{2+} (from left to right: 0, 2, 5, and 10 μM, respectively) for 30 min. Excitation at 543 nm for red emission of **HN7-S** (650-720 nm).

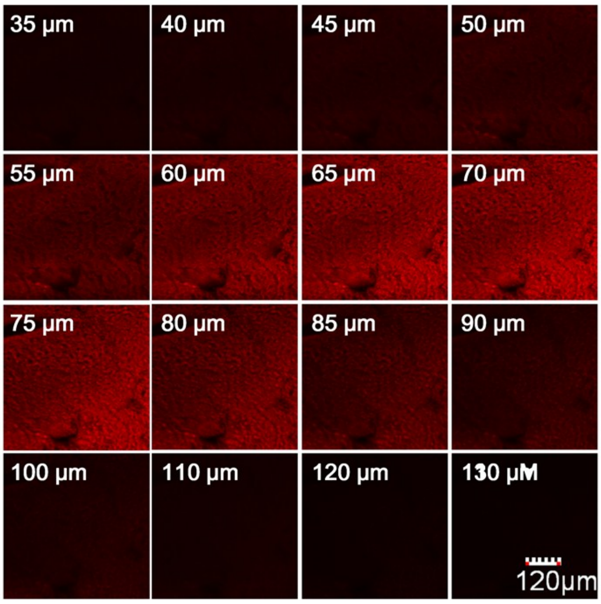

Figure S15. Depth fluorescence images of **HN7-S** for Hg^{2+} detection in tissues were obtained with spectral confocal microscopy (Olympus, FV1000). The changes of fluorescence intensity with scan depth were determined by spectral confocal microscopy (Olympus, FV1000) in the z-scan mode (from 0 to 150 μm; step size: 1 μm). The images were collected at 650-720 nm (red channel). The slice was cultured with **HN7-S** (5 μM) for 60 min and then Hg^{2+} (10 μM) for another 60 min.

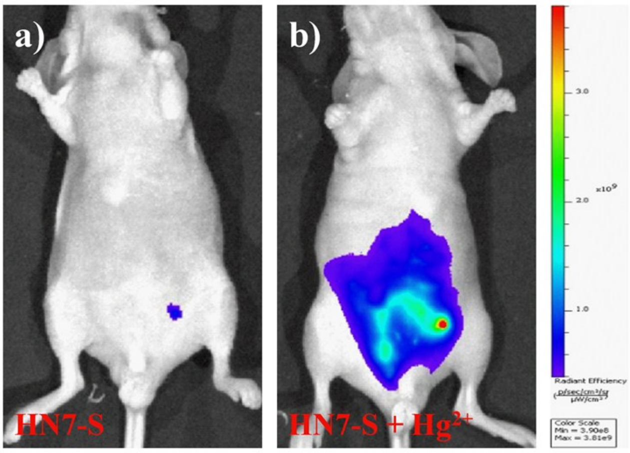

Figure S16. Fluorescent images of mice (pseudocolor). The mice were imaged using a Caliper VIS Lumina XR small animal optical *in vivo* imaging system with an excitation filter 605 nm and a Cy5.5 emission filter. Left: Imaging of control group after intraperitoneal injection of **HN7-S** probe (20 nanomoles) for 30 min; Right: intraperitoneal injection of **HN7-S** probe (20 nanomoles) for 30 min, followed by intraperitoneal injection of Hg^{2+} (20 nanomoles). The mice were imaged 30min after Hg^{2+} injection.

Table S5. Analytical data comparison of the probe with reported rhodamine-based NO probes.

Probe	λ_{abs}/nm	λ_{em}/nm	SS[c]	Detection Limit/nM	Bio-imaging
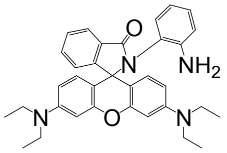 3	554	574	20	3	-
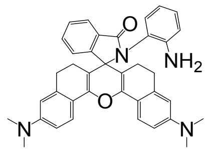	600	676	76	8	HeLa cell, Living Mice

[a]The maximal absorption of the dye. [b]The maximal emission of the dye. [c]Stokes shift.

Table S6. Analytical data comparison of the probe with reported rhodamine-based Hg^{2+} probes.

probe	λ_{abs}/nm	λ_{em}/nm	SS[c]	Detection Limit/nM	Bio-imaging
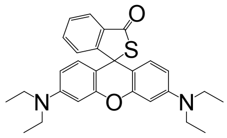 4	560	585	25	20	-
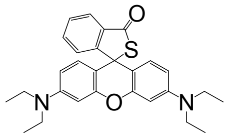 5	559	585	26	2.1	-
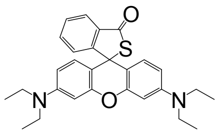 6	-	-	-	-	Arabidopsis thaliana
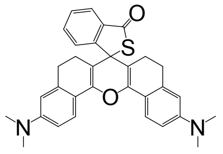	600	678	78	3	HeLa cell, Living Mice

[a]The maximal absorption of the dye. [b]The maximal emission of the dye. [c]Stokes shift.

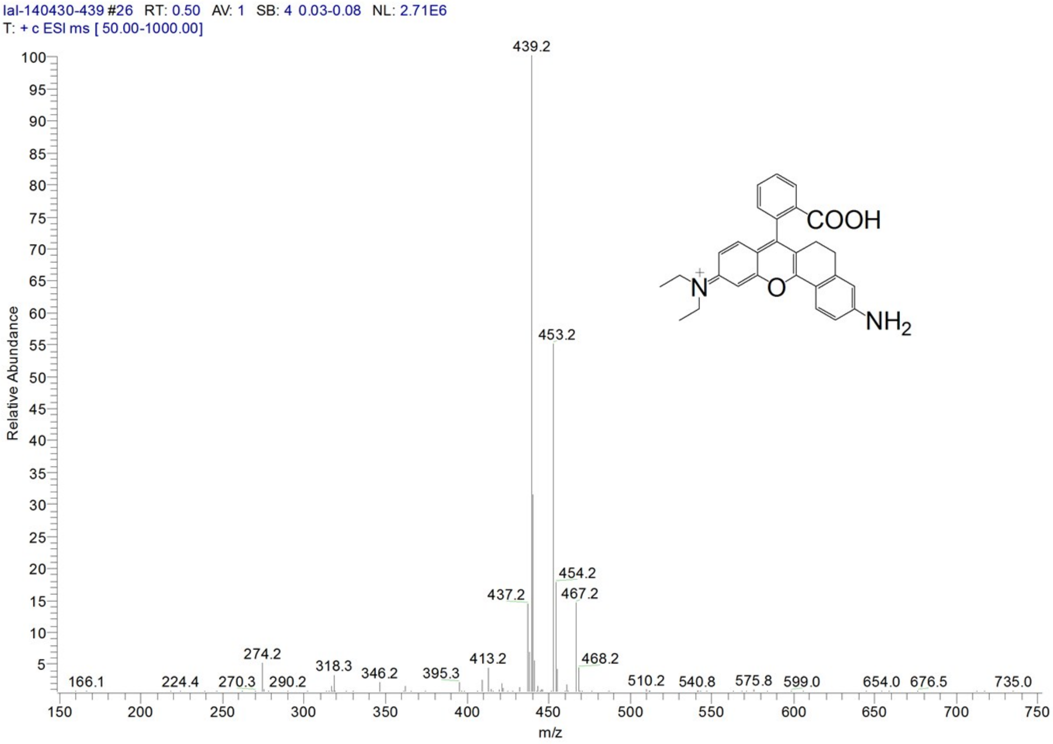

Mass spectra of **HN2**.

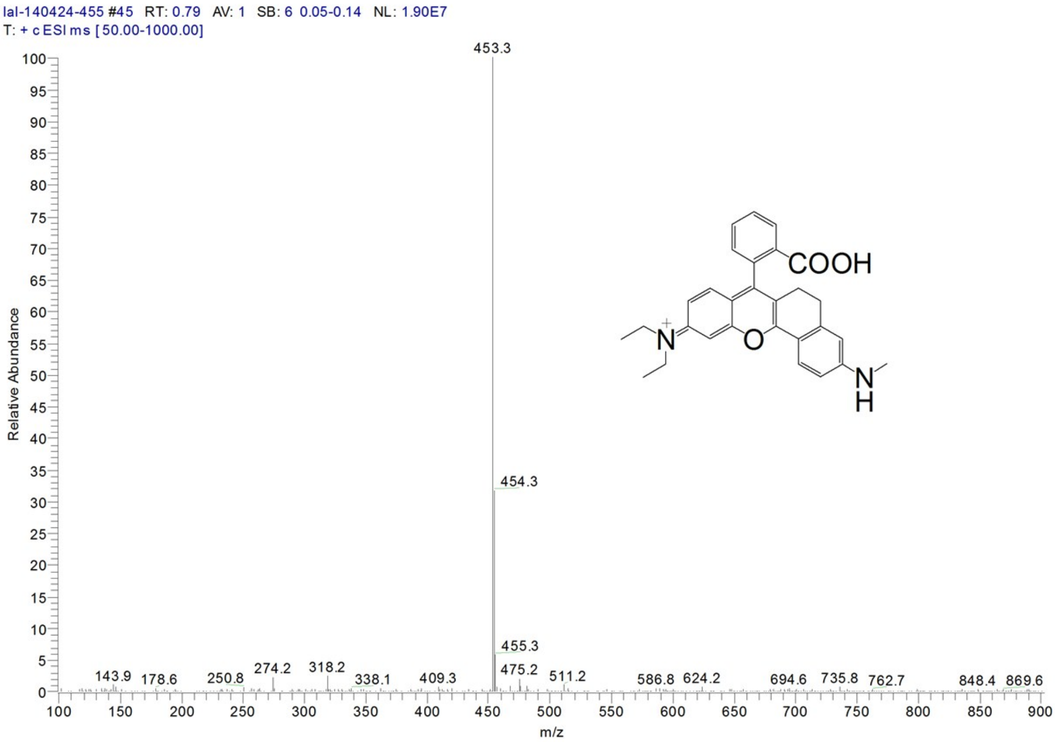

Mass spectra of **HN4**.

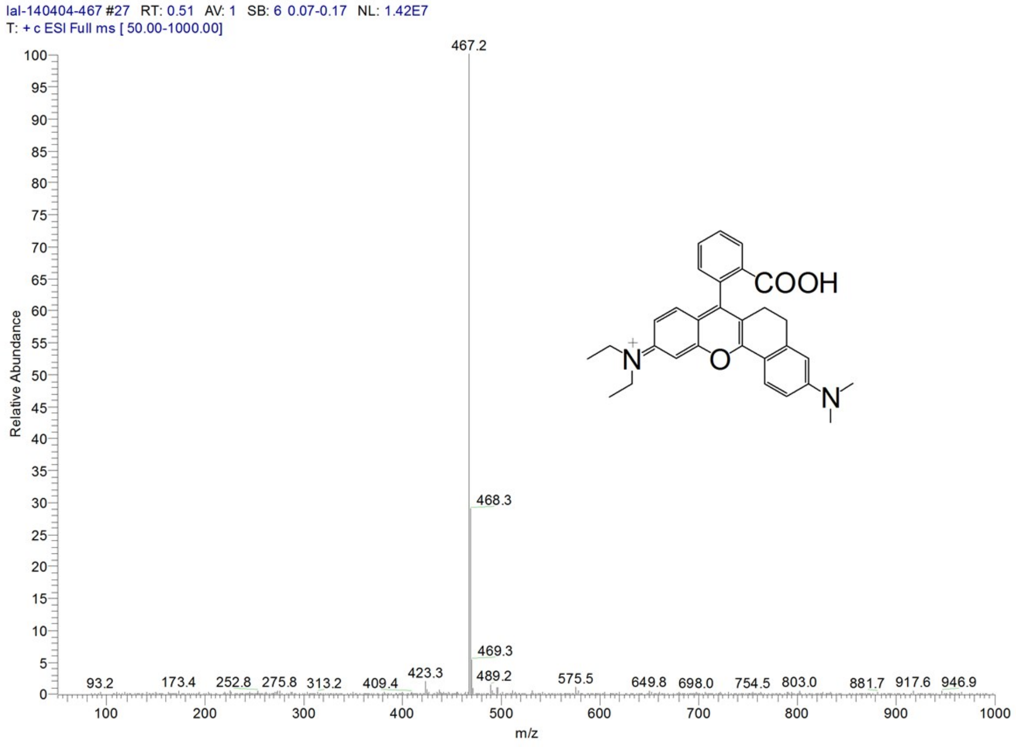

Mass spectra of **HN6**.

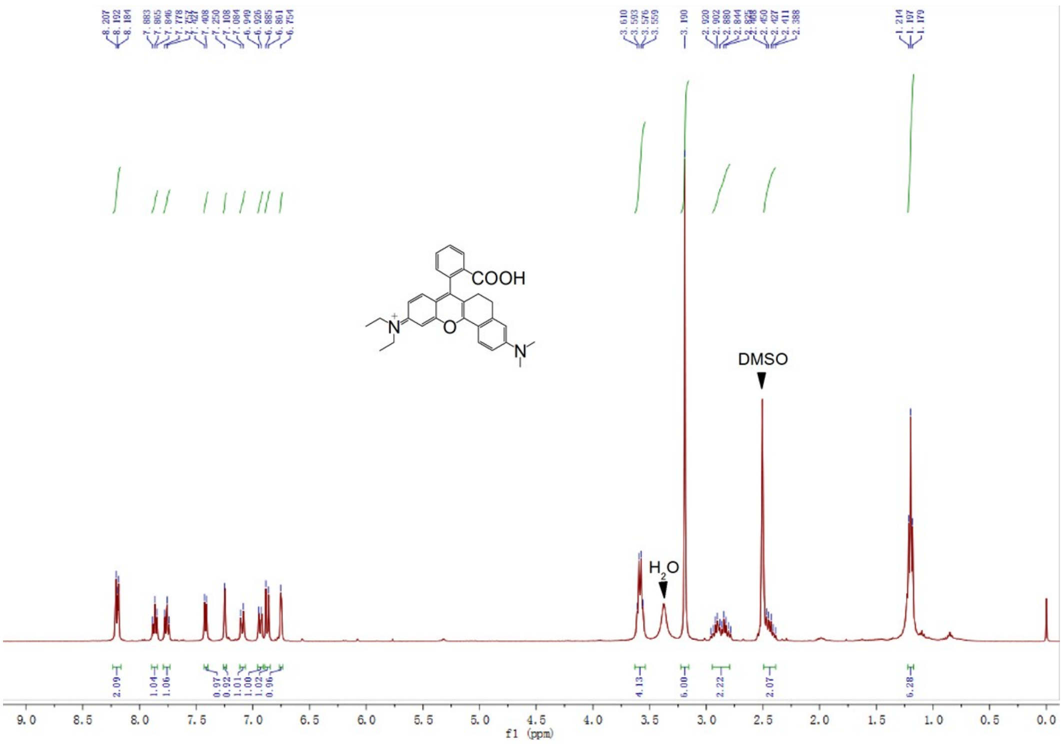

^{1}H NMR spectra of **HN6**.

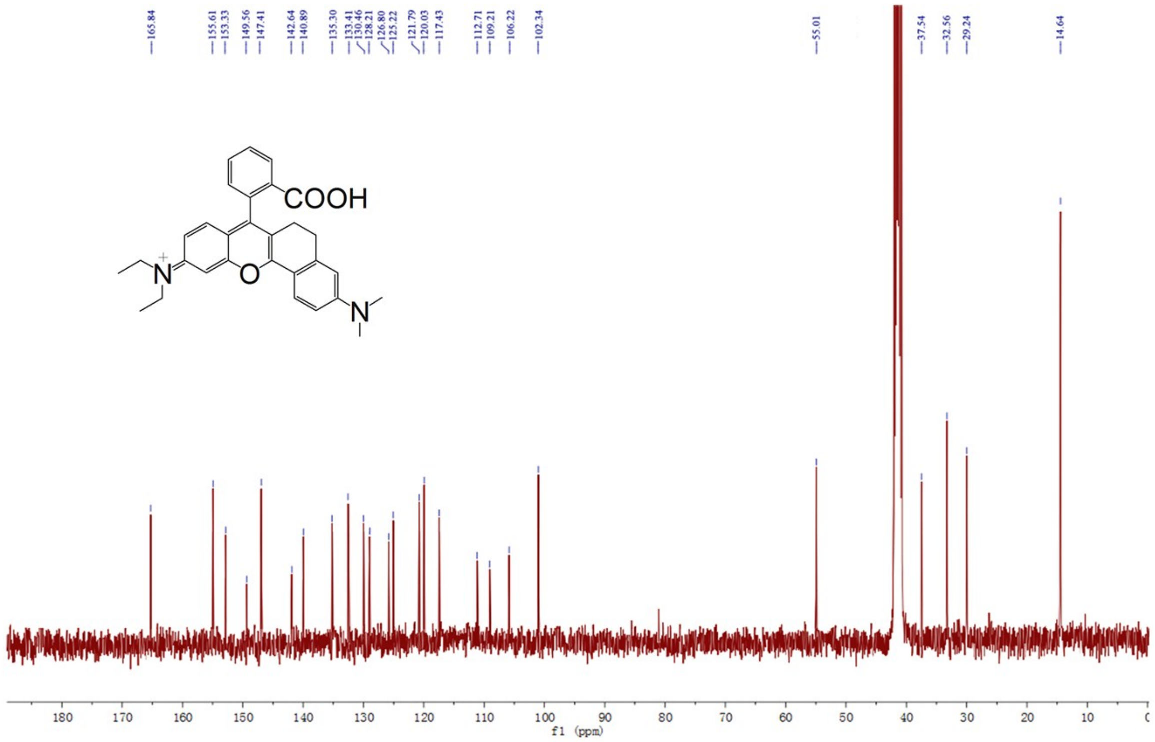

^{13}C NMR spectra of **HN6**.

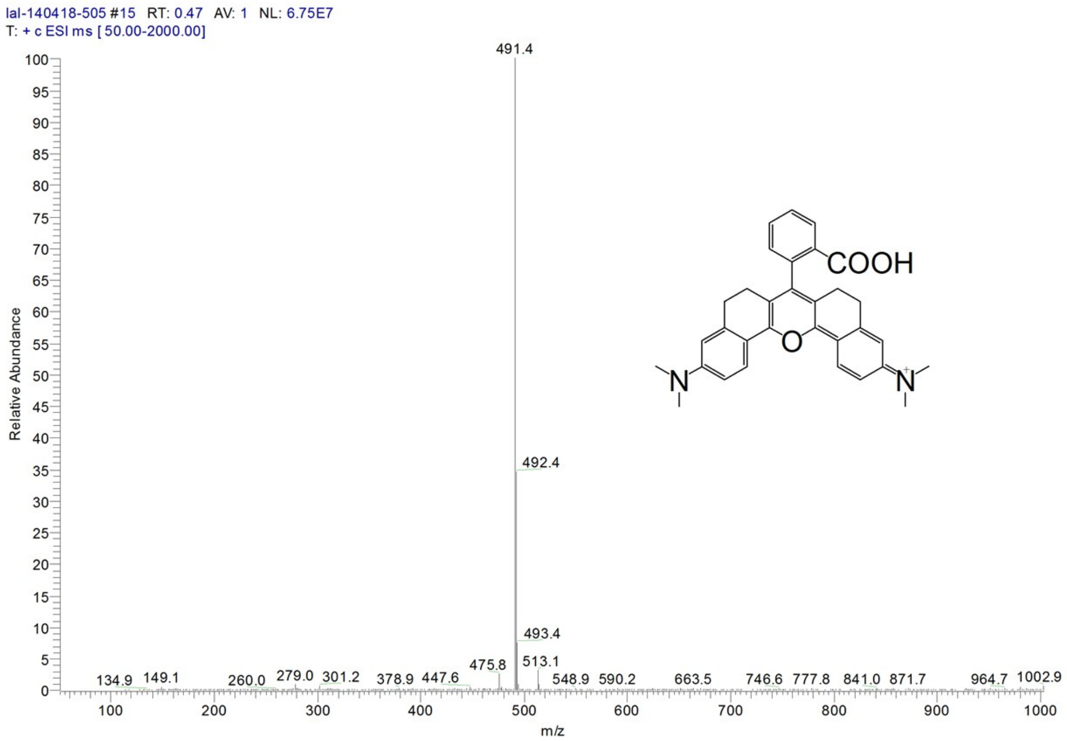

Mass spectra of **HN7**.

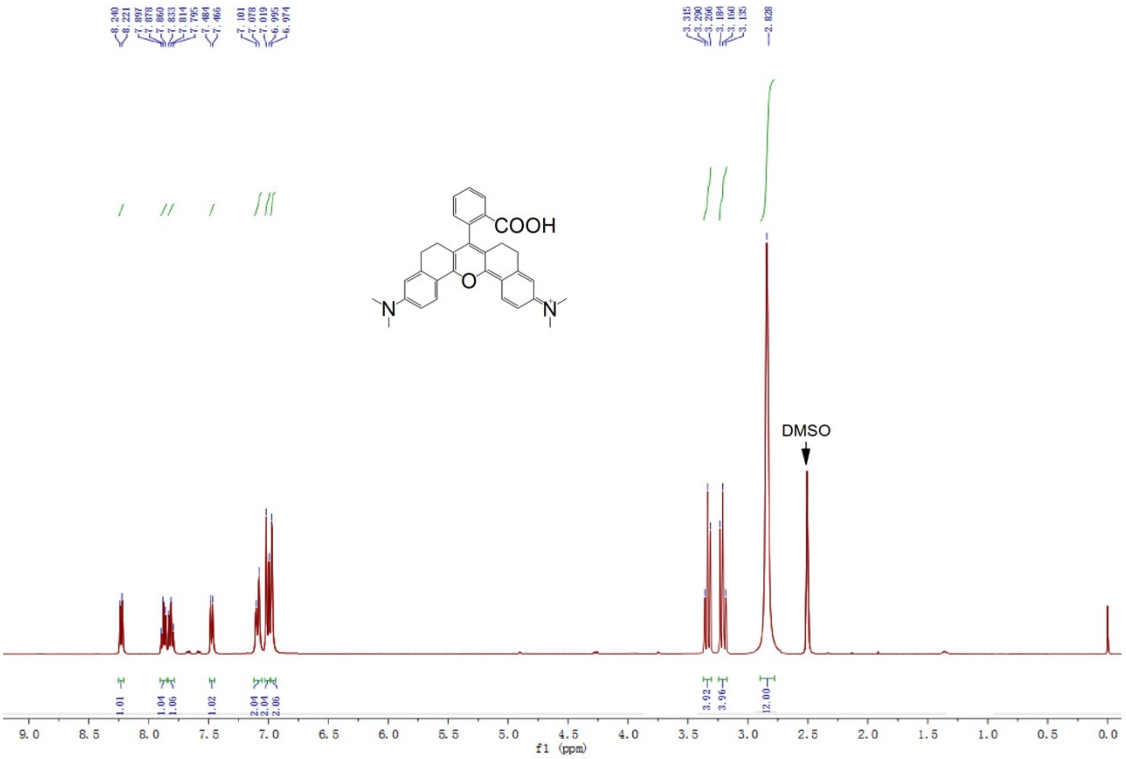

^{1}H NMR spectra of **HN7**.

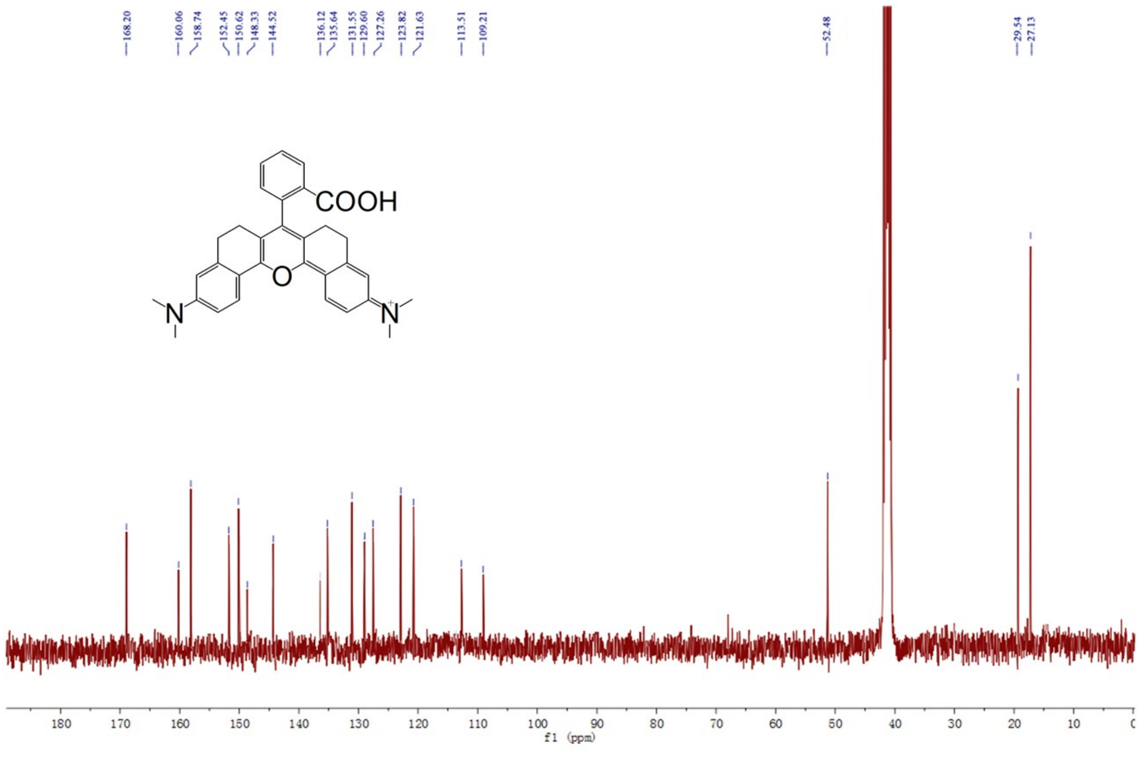

^{13}C NMR spectra of **HN7**.

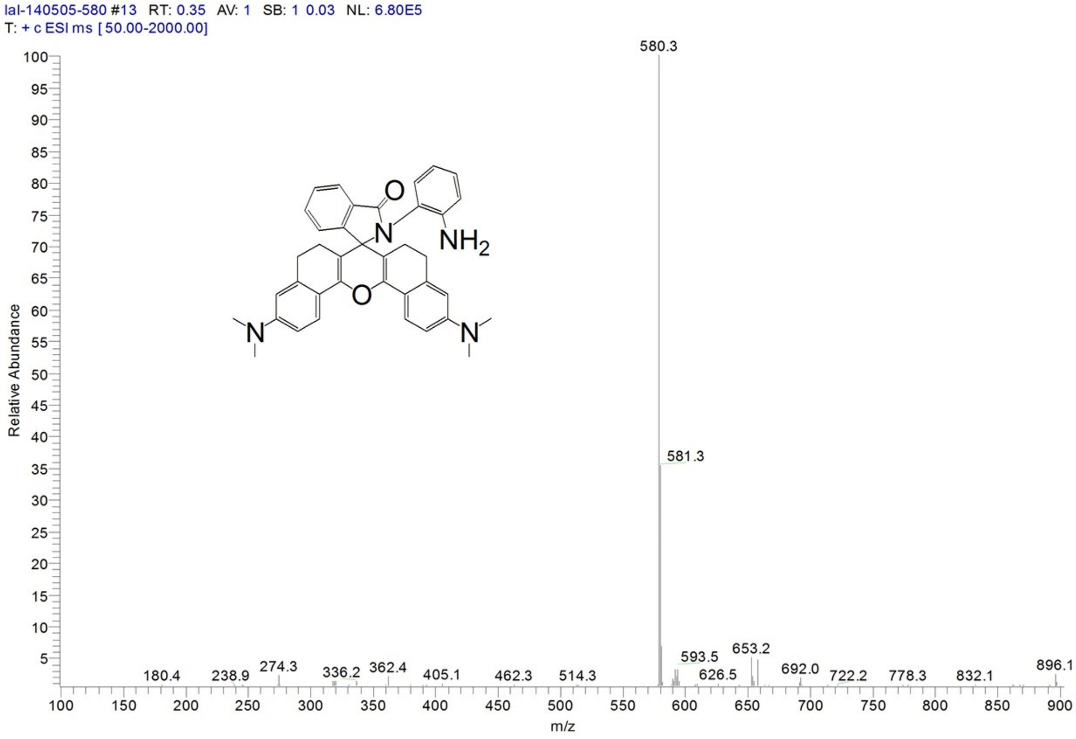

Mass spectra of **HN7-N2**.

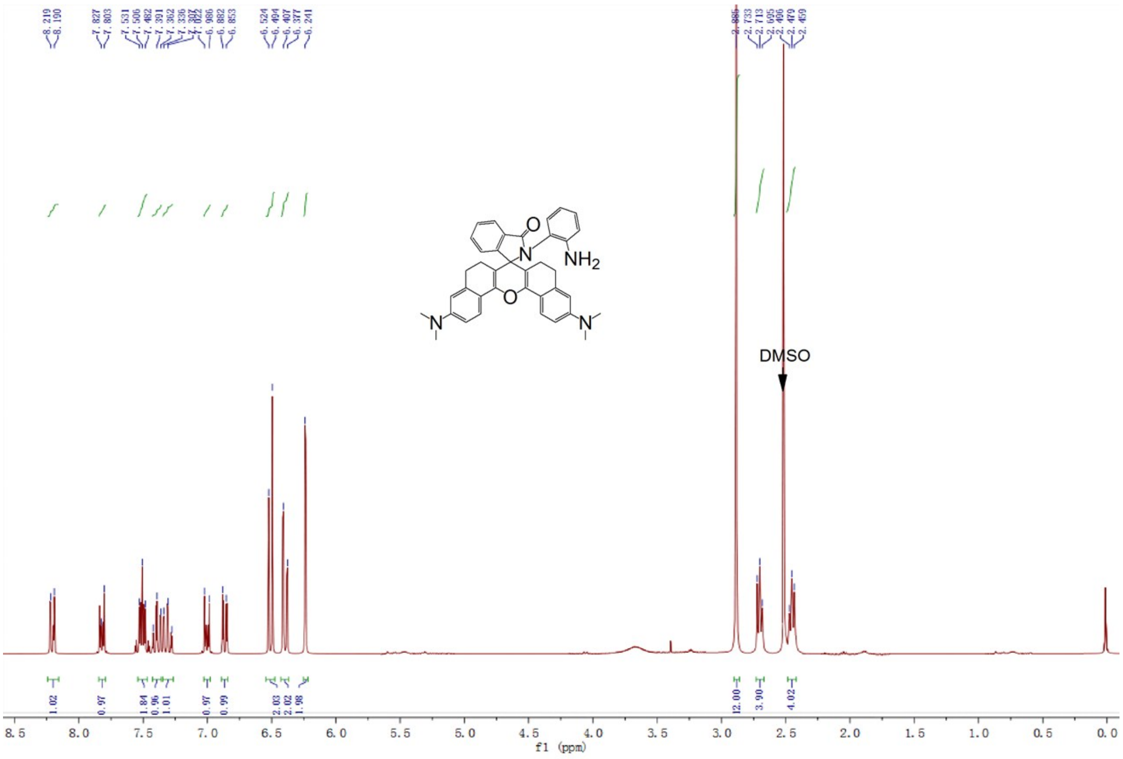

^{1}H NMR spectra of **HN7-N2**.

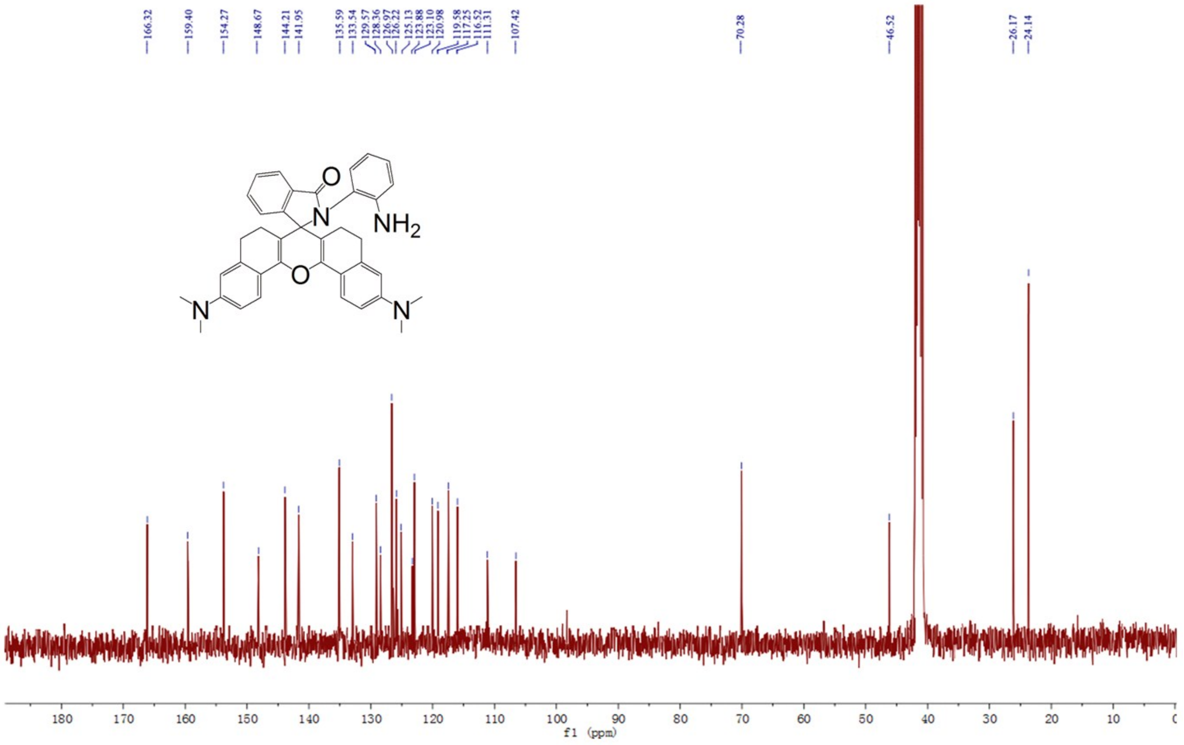

^{13}C NMR spectra of **HN7-N2**.

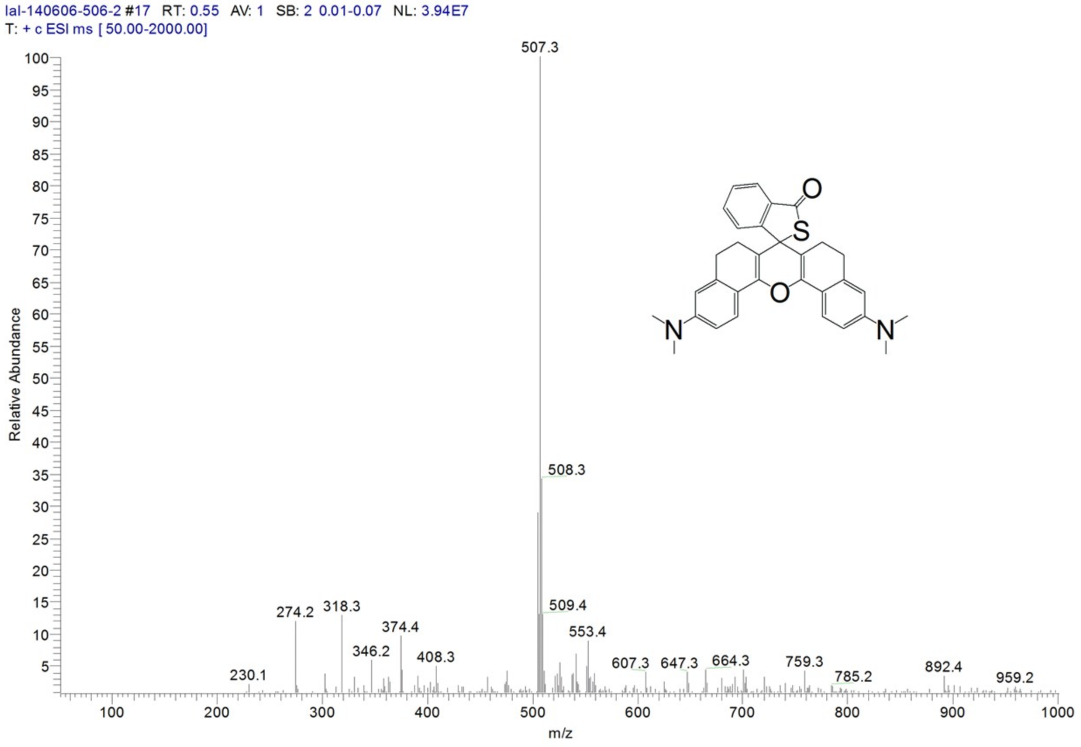

Mass spectra of **HN7-S**.

References

[1] B. Valeur, Molecular Fluorescence: Principles and Applications, Wiley-VCH, 2001.

[2] D. Oushiki, H. Kojima, T. Terai, M. Arita, K. Hanaoka, Y. Urano and T. Nagano, *J. Am. Chem. Soc.*, 2010, **132**, 2795-2801.

[3] H. Zheng, G. Shang, S. Yang, X. Gao and J. Xu, *Org. Lett.*, 2008, 10, 2357-2360.

[4] W. Shi and H. Ma, *Chem. Commun.*, 2008, **16**, 1856-1858.

[5] X. Zhan, Z. Qian, H. Zheng, B. Su, Z. Lan and J. Xu, *Chem. Commun.*, 2008, **16**, 1859-1861.

[6] Y. Zhang, W. Shi, D. Feng, H. Ma, Y. Liang and J. Zuo, *Sens. Actuators B: Chem.*, 2011, 153, 261-265.
